# Impact of prior antiplatelet therapy on safety and efficacy of alteplase in acute ischemic stroke: a systematic review and meta-analysis

**DOI:** 10.1007/s10072-025-08024-x

**Published:** 2025-02-22

**Authors:** Ahmed Naeem, Hesham Kelani, Hazem Mohamed Salamah, Rowan H. Elhalag, Hossam Tharwat Ali, Ayham Mohammad Hussein, Omar Abdelnasser, Mostafa Mahmoud Naguib, Salem Elshenawy, Abdelrhman M. Abdelwahab, Nadia Albaramony, Omar El Sayed Rageh, Abdallah R. Allam, Aliaa Bakr, Mohamed Abuelazm, Ahmed Madkoor, Travis R. Quinoa, Arthur D. Kay, David P. Lerner, Lisa R. Merlin, Eytan Raz, Alejandro M. Spiotta, Stephan A. Mayer

**Affiliations:** 1 Al-Azhar Faculty of Medicine, Asyut, Egypt; 2 Neurology Department, SUNY Downstate Health Science University at One Brooklyn Health, New York City, NY USA; 3 Faculty of Medicine, Zagazig University, Zagazig, Egypt; 4 Faculty of Medicine, Alexandria University, Alexandria, Egypt; 5 Qena Faculty of Medicine, South Valley University, Qena, Egypt; 6 Faculty of Medicine, Al-Balqa’ Applied University, Salt, Jordan; 7 Faculty of Medicine, Ain Shams University, Cairo, Egypt; 8 Faculty of Medicine, Al-Azhar University, Damietta, Egypt; 9 Neurology and Neurocritical Care Department, Mayo Clinic, Jacksonville, FL USA; 10 Faculty of Medicine, Tanta University, Tanta, Egypt; 11 Faculty of Medicine, Menoufia University, Menoufia, Egypt; 12 Al-Azhar Faculty of Medicine for Girls, Cairo, Egypt; 13 Burke Neurological Institute, Weill Cornell Medical College, New York City, NY USA; 14 Department of Neurosurgery, Rutgers New Jersey School of Medicine, Newark, NJ USA; 15 Department of Neurosurgery, NYU Langone Health, New York City, NY USA; 16 Neurosurgery Department, Medical University of South Carolina, Charleston, SC USA; 17 Westchester Medical Center Health Network, Departments of Neurology and Neurosurgery, New York Medical College, Valhalla, NY USA; 18 Departments of Neurology, Physiology, and Pharmacology, SUNY Downstate Health Sciences University, Brooklyn, NY USA

**Keywords:** IV thrombolysis, Acute ischemic stroke, Prior antiplatelets, Alteplase, Systematic review, Meta-analysis

## Abstract

**Background:**

Intravenous thrombolysis (IVT), utilizing the clot-dissolving medications alteplase (rt-PA) or tenecteplase (TNK), is the cornerstone in acute ischemic stroke (AIS) emergency intervention. However, the impact of prior antiplatelet therapy (APT) on post-IVT outcomes when utilizing alteplase remains controversial. We conducted a systematic review and meta-analysis to evaluate the effect of prior APT on the outcomes after using alteplase in AIS patients.

**Methods:**

We conducted a systematic review and meta-analysis synthesizing studies, which were retrieved by systematically searching PubMed, Web of Science, SCOPUS, and Cochrane through June 30, 2024. We used the R language V. 4.3. to pool dichotomous data using odds ratio (OR) with a 95% confidence interval (CI). PROSPERO ID: CRD42024495393.

**Results:**

Thirty studies were included in our analysis, with 436,232 patients. Prior APT was significantly associated with increased odds of symptomatic intracranial hemorrhage (sICH) (OR, 1.78; 95%CI [1.48, 2.13]; *P* < 0.01), any ICH (OR, 1.44; 95%CI [1.16, 1.78]; *P* < 0.01), mortality (OR, 1.39; 95%CI [1.23, 1.58]; *P* < 0.01), and poor functional outcomes (modified Rankin Scale score of 3–6 [mRS 3–6]) (OR, 1.81; 95%CI [1.03, 3.19]; P = 0.04). Additionally, prior APT significantly reduced the odds of good functional outcome [mRS 0–2] (OR, 0.85; 95%CI [0.74, 0.97]; P = 0.02).

**Conclusion:**

Prior APT increased hemorrhagic complications, mortality, and poor functional outcome, while reducing the odds of good functional outcome after IV alteplase. Future research should focus on identifying adjunctive agents that may decrease hemorrhagic complications and investigate the impact of various APT regimens and alternative thrombolytics beyond alteplase in this specific population.

**Supplementary Information:**

The online version contains supplementary material available at 10.1007/s10072-025-08024-x.

## Introduction

Prompt reperfusion is crucial in acute ischemic stroke (AIS) to minimize brain damage and maximize patient recovery [1, 2]. The primary therapeutic objective revolves around expeditiously reinstating blood circulation to the affected cerebral region by reopening the occluded artery [3]. Intravenous thrombolysis (IVT), utilizing the clot-dissolving medications alteplase (rt-PA) or tenecteplase (TNK), is the cornerstone in AIS emergency intervention [4–6]. The effectiveness of IVT is contingent on time, with a short treatment window of 4.5 h from the onset of stroke symptoms, owing to an increased risk of hemorrhage outside this window [7, 8].

Patient comorbidities and baseline medications may also affect IVT post-treatment outcomes [9].

Prior exposure to antiplatelet therapy (APT) such as aspirin or clopidogrel is commonly encountered in AIS patients treated with IVT, occurring in up to 40% [10, 11]. While current guidelines do not consider APT an absolute contraindication to IVT in eligible patients, its impact on post-thrombolytic outcomes remains controversial [12, 13].

The existing literature on the impact of prior APT on the outcomes of AIS patients treated with IV alteplase is notably inconsistent. Some studies have suggested potential drawbacks of pre-stroke APT on post-thrombolytic safety and efficacy [14]. Conversely, other studies have found no significant effect of prior APT on post-thrombolytic outcomes of AIS patients [10]. Additionally, a third body of research reports an increased risk of antiplatelet-associated symptomatic intracerebral hemorrhage (sICH) alongside improved functional outcomes and early recanalization after IV alteplase in patients on APT [15, 16]. Recently, an analysis of 321,819 patients reported that prior APT was associated with an increased risk of sICH and decreased odds of achieving a modified Rankin Scale score of 0–2. However, sICH rates were comparable to those observed in landmark trials, which may indicate a general clinical benefit of IV alteplase in AIS patients with prior APT [17].

Given the conflicting evidence, we conducted a systematic review and meta-analysis to comprehensively evaluate the impact of prior APT on outcomes after IV alteplase in AIS patients.

## Methods

This study was registered in PROSPERO (CRD42024495393) and prepared in accordance with the Preferred Reporting Items for Systematic Reviews and Meta-analyses (PRISMA) and the Meta-analysis of Observational Studies in Epidemiology (MOOSE) reporting guidelines [18, 19]. PRISMA statement checklist 2020 is outlined in (Table S1).

### Search strategy

The following databases were systematically searched: Web of Science, SCOPUS, PubMed (MEDLINE), and Cochrane Central Register of Controlled Trials (CENTRAL) on June 30, 2024. The following keywords and their MeSH terms were used to formulate the search strategy: “stroke”, “thrombolysis”, “antiplatelet”, and “prior”. The detailed search approach is presented in (Table S2).

### Eligibility criteria

The inclusion criteria encompassed randomized control trials (RCTs) and controlled observational studies, including cross-sectional, prospective, or retrospective cohort, and case–control studies published in English, which evaluated the possible impact of prior APT on IV alteplase in patients diagnosed with AIS. Studies were excluded if they were secondary research articles (systematic reviews, meta-analyses, and editorials), case reports; case series, animal-based studies, or studies not reporting any of our outcomes of interest. Primary outcomes of interest were sICH, any ICH, mortality at discharge, good functional outcome (modified Rankin Scale score of 0–2), and poor functional outcome (modified Rankin Scale score of 3–6). The modified Rankin Scale levels 0–2 suggest minimal impairment or none at all, whereas levels 3–6 indicate escalating levels of impairment and reliance on others, with level 6 denoting the most critical outcome of death.

### Study selection process

The results from the databases were imported into Rayyan.ai, where duplicates were automatically identified and eliminated. Title and abstract screening was carried out using Rayyan.ai for the remaining results to assess their relevance to the meta-analysis. Studies deemed relevant were exported to a Microsoft Excel sheet, and their full-text papers were obtained for further evaluation in the full-text screening phase, adhering to our inclusion criteria. Throughout all screening phases, four reviewers worked independently with conflicts resolved by a fifth reviewer [20].

### Data extraction

For the baseline characteristic, a prespecified Excel sheet was used by four authors independently to extract the following data from the eligible studies: author’s name, study design, country of study origin, number of participants, age of participants, gender of participants, medical comorbidities as; (hypertension, diabetes, atrial fibrillation, and hyperlipidemia), prior APT use, previous stroke or transient ischemic attack (TIA), baseline National Institute of Health Stroke Scale (NIHSS), IVT agent and dose, onset-to-IVT time, EVT utilization, the definition of sICH, smoking status, and duration of follow up. Safety and efficacy outcomes were extracted in the form of events and total for each treatment group.

### Quality assessment

The risk of bias in individual studies was assessed independently by three authors using the Risk of Bias in Non-Randomized Studies of Interventions (ROBINS-I) tool [21], and conflicts were resolved by the first author.

### Statistical analysis

Data were analyzed using the R statistical programming language [22]. Dichotomous data were presented as odds ratio (OR) with the corresponding 95% confidence interval (CI), and a meta-analysis with the Mantel–Haenszel method was performed for each outcome. A p-value of < 0.05 was identified for statistical significance. Heterogeneity was assessed using the tau squared and I^2^ statistic, with I^2^ ≥ 60% indicating a substantial heterogeneity. Sensitivity analysis (leave-one-out test and subgroup analysis) was used to resolve any heterogeneity if detected. Egger’s test and funnel plot asymmetry method were used to assess publication bias.

For all outcomes, subgroup analyses based on study design, mean baseline NIHSS scores, and time window from onset to treatment were conducted whenever possible. For sICH, additional subgroup analysis was performed based on different sICH definitions.

## Results

### Literature search and screening

**Fig. 1:**
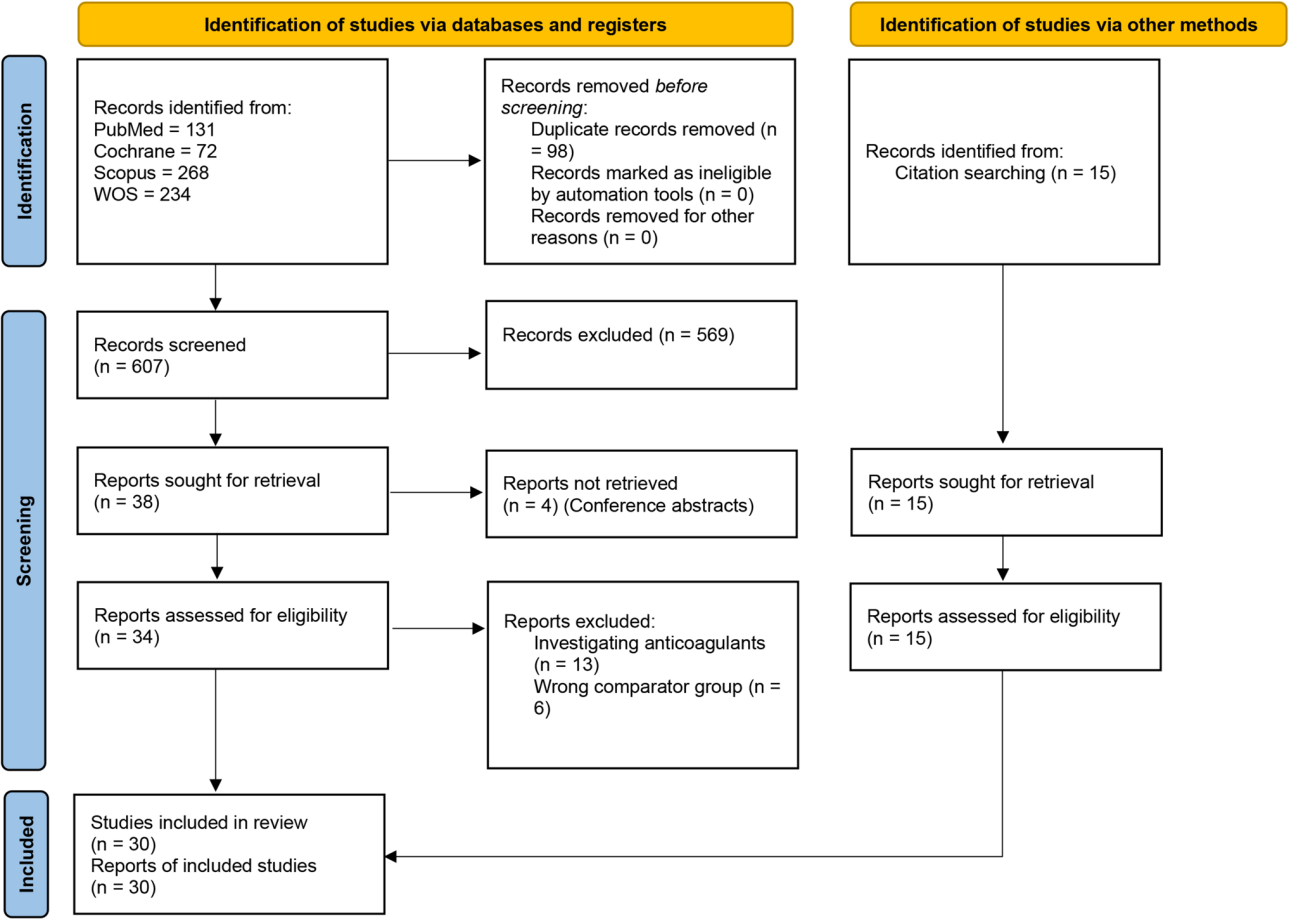
PRISMA flow diagram of the screening process

Searching the four databases yielded 705 articles. After the removal of 98 duplicates, 607 records went through the title and abstract screening phase. Subsequently, 34 studies entered the full-text screening phase, with 15 studies found to fulfill the inclusion criteria. We identified an additional 15 studies through manual search by screening the references of included studies to end up with a total of 30 studies included in our analysis (Fig. 1). References of the included studies are shown in Table S3.

### Characteristics of the included studies

Thirty studies were included in our analysis representing 436,232 participants (188,402 with prior APT and 247,830 without prior APT). The mean age of participants ranged from 62.8 to 75 years. Alteplase was the thrombolytic agent used across all studies. Notably, data about specific types of APT and duration were limited. Baseline characteristics of the included studies are outlined in (Table 1).

**Table 1:** Baseline characteristics of included studies

Study	Study design	Country	IVT			Onset to IVT, min	sICH definition	Prior APT	No. of patients	Female sex, %	Age, y, mean(SD)	
			Agent	Dose	Time window							
Robinson 2017 (ENCHANTED)	Subgroup analysis of RCT	Multinational	IV Alteplase	(45.7 ± 14) mg	4.5 h	161.5 [116 - 210]	SITS-MOST, ECASS-2, ECASS-3, NINDS, and IST-3	Yes	752	39	71 (11)	
						173 [130 - 220]		No	2533	38	65 (13)	
Dharmasaroja 2011	Observational	Thailand	IV Alteplase	0.9 mg/kg	4.5 h	154 (42)	ECASS-2	Yes	52	40	64 (13)	
								No	140			
Cucchiara 2009	Observational	Multinational	IV Alteplase	NA	3 h	145 (33)	ECASS-2	Yes	337	43	68 (13)	
								No				
Lindley 2015 (IST 3)	Subgroup analysis of RCT	Multinational	IV Alteplase	0.9 mg/kg, maximum dose of 90mg	6 h	252 (72)	IST-3	Yes	775	52	NA	
								No	740			
Strbian 2012	Observational	Finland and Switzerland	IV Alteplase	0.9 mg/kg	4.5 h	120 (100)	ECASS-2	Yes	413	45.7	70 (25)	
								No	561			
Tanne 2002	Observational	Multinational	IV Alteplase	NA	3 h	NA	NINDS	Yes	386	43.8	66.5 (13.7)	
								No	813			
Hack 1998 (ECASS-2)	Subgroup analysis of RCT	Multinational	IV Alteplase	0.9 mg/kg, maximum dose of 90 mg	6 h	NA	ECASS-2	Yes	84	39.4	68	
								No	325			
Bluhmki 2009 (ECASS-3)	Subgroup analysis of RCT	Multinational	IV Alteplase	0.9 mg/kg, maximum dose of 90 mg	4.5 h	239 [225 - 255]	NINDS	Yes	130	37	64.9 (12.2)	
								No	288			
NINDS 1995	Subgroup analysis of RCT	USA	IV Alteplase	0.9 mg/kg	3 h	240 [225 - 255]	NINDS	Yes	127	27.2	68 (11)	
								No	185			
Watson-fargie 2015	Observational	UK	IV Alteplase	NA	NA	161.3 (57.7)	NINDS, ECASS-2	Yes	132	56	70.1 (13.6)	
								No	216			
Xian 2016	Observational	USA	IV Alteplase	0.9 mg/kg	4.5 h	138 [108 - 170]	ECASS-2	Yes	38844	49.7	73.7 (13.2)	
						138 [107 - 172]		No	46228	51.4	67.2 (15.7)	
Mowla 2021	Observational	USA	IV Alteplase	0.9 mg/kg	4.5 h	NA	ECASS-2	Yes	360	47.8	74.3 (13.3)	
								No	463	50.1	68.7 (16.1)	
Frey 2020	Subgroup analysis of RCT	Germany	IV Alteplase	0.9 mg/kg	4.5 h	186 [150 - 228]	SITS-MOST	Yes	75	36.6	70.3 (8.2)	
								No	86	34.8	62.8 (12.1)	
Uyttenboogaart 2008	Observational	Netherlands	IV Alteplase	NA	4.5 h	165 [30 - 270]	SITS-MOST	Yes	89	78	73 (11)	
						175 [70-285]		No	212	47	66 (15)	
Couture 2021	Observational	France	IV Altelplase	NA	NA	152.4 (47.8)	HBC	Yes	74	47	72.4 (11.7)	
						155.7 (45.2)		No	133	46	67.5 (14.7)	
Hermann 2009	Observational	Germany	IV Alteplase	NA	3 h	138 (34)	SITS-MOST	Yes	39	45	71 (11)	
								No	24		67 (11)	
Lin 2021	Observational	Taiwan	IV Alteplase	(0.77 ± 0.15) mg/kg	3 h	122.4 (58)	NINDS, ECASS-2	Yes	277	38	75 (8.2)	
						120.8 (56.6)		No	825	39.5	74.9 (8.6)	
Ibrahim 2010	Observational	Canada	IV Alteplase	NA	3 h	140 (34)	SITS-MOST	Yes	104	51.6	71.2 (10.2)	
								No	180	78	68.3 (13.9)	
Chen 2016	Observational	China	IV Alteplase	0.9 mg/kg, maximum dose of 90 mg	6 h	188.7 (48.4)	ECASS-2	Yes	23	39.1	70 (8.7)	
								No	122	34.4	63.2 (11.8)	
Hang Jing 2020	Observational	China	IV Alteplase	0.9 mg/kg	3 h	177.9 (56.7)	ECASS-2	Yes	23	27	74.5 (7.7)	
						170.1 (58.1)		No	36	34	68.8 (10.6)	
Dorado 2010	Observational	Spain	IV Alteplase	NA	NA	145.5 (38)	ECASS-2	Yes	72	37.4	70.5 (9.3)	
								No	162		66.9 (13.3)	
Bravo 2008	Observational	Spain	IV Alteplase	(67 ± 11.6) mg	3 h	148.5 (29.7)	ECASS-2	Yes	137	35	72.2 (7.2)	
						151.1 (56.5)		No	468	43.8	66.6 (11.2)	
Tsivgoulis 2018	Observational	Greece	IV Alteplase	NA	NA	154 (56)	SITS-MOST, ECASS-2, and NINDS	Yes	1043	31.6	70.2 (10.9)	
						155 (57)		No	1043	32.1	70 (11.4)	
Pan 2015	Observational	China	IV Alteplase	(0.88 ± 0.05) mg/kg	4.5 h	168 [132 - 198]	SITS-MOST, ECASS-2, and NINDS	Yes	157	43.7	66 (10.1)	
						168 [132 - 198]		No	951	38.2	62.7 (11.2)	
Choi 2016	Observational	Korea	IV Alteplase	27%: (0.6 mg/kg), 73%: (0.9 mg/kg)	4.5 h	NA	ECASS-2	Yes	324	40	70.2 (10.9)	
				19.7%: (0.6 mg/kg), 80.3%: (0.9 mg/kg)				No	324	41.3	70 (11.4)	
Sanak 2012	Observational	Czech Republic	IV Alteplase	NA	3 h	NA	ECASS-2	Yes	56	50	69.8 (9.8)	
								No	90	43.3	65.8 (12.5)	
Meseguer 2015	Observational	France	IV Alteplase	NA	NA	160 [130 - 181]	ECASS-2	Yes	288	43.4	74·5 (13·5)	
						160 [120 - 185]		No	586	46	65·8 (16·9)	
Diedler 2010	Observational	Germany	IV Alteplase	NA	3 h	140 (51)	SITS-MOST, ECASS-2, and NINDS	Yes	3782	35.1	71 (12)	
						140 (50)		No	7954	41.2	65 (12)	
Peng 2024	Observational	USA	IV Alteplase	NA	4.5 h	135(54.2)	NINDS	Yes	139٬475٫00	42.14	68.6(15.1)	
								No	182٬344٫00	50.4		

Study	Prior APT Agent(s), n		Previous stroke or TIA, %	HTN, %	Diabetes, %	Current smoking, %	AF, %	Hyperlipidemia, %	NIHSS		EVT, %	Follow-up time
									mean (SD)	median [IQR]		
Robinson 2017 (ENCHANTED)	NA	NA	33	78	26	16	29	37	9 (6.6)	8 [5 - 14]	75	3 months
	-	-	14	75	18	26	16	11			71.7	
Dharmasaroja 2011	NA	NA	8.1	61.4	25.3	NA	24.3	29.9	17.3 (27.6)	11 [2 - 39]	NA	3 months
	-	-										
Cucchiara 2009	SAPT	294	13.2	71.6	20.2	22.2	27.8	NA	14	14	NA	3 months
	DAPT	43										
	-	-										
Lindley 2015 (IST 3)	Aspirin	639	NA	64	12	NA	31	NA	NA	NA	NA	6 months
	Clopidogrel	69										
	Dipyridamole	66										
	-	-										
Strbian 2012	Aspirin	318	12.5	58.9	14.1	NA	NA	38	10 (13.3)	10 [1 - 19]	NA	3 months
	Aspirin and Dipyridamole	48										
	Clopidogrel	19										
	-	-										
Tanne 2002	Aspirin	360	13	60.7	20	28.3	21.3	NA	NA	NA	NA	NA
	-	-										
Hack 1998 (ECASS-2)	Aspirin	84	19.3	52.8	21.3	19.3	21.8	NA	NA	11	NA	3 months
	-	-										
Bluhmki 2009 (ECASS-3)	NA	NA	NA	62	15	31	53	NA	10.7 (5.6)	9 [6 - 15]	NA	3 months
	-	-										
NINDS 1995	Aspirin	41	NA	65.7	21.8	NA	18.6	33.3	NA	14	NA	3 months
	-	-										
Watson-fargie 2015	NA	NA	20	54	15	45	24	NA	11.2 (6.7)	NA	NA	NA
	-	-										
Xian 2016	Aspirin	15116	36.7	82.1	32.2	13.8	25.7	54.4	10.4 (8.4)	10 [5 - 17]	0	At discharge
	Aspirin and Clopidogrel	2397							31.9			
	Clopidogrel	2037										
	Aspirin and Dipyridamole	393										
	-	-	15.8	66	21.9	20.7	16.7	31.9		10 [5 - 16]		
Mowla 2021	Aspirin (81mg)	191	26.9	86.9	24.7	15.8	26.1	NA	11.5 (6.4)	NA	NA	NA
	Aspirin (325mg)	58										
	Clopidogrel (75mg)	45										
	Aspirin and Dipyridamole	15										
	Aspirin and Clopidogrel	51										
	-	-	8.9	64.8	21.6	19.9	17.5					
Frey 2020	Aspirin	134	31.7	73.2	26.8	58	16.5	59.8	6.8 (5.3)	5 [4 - 9]	NA	3 months
	Clopidogrel	34										
	Dipyridamole	6										
	Triflusal	1										
	DAPT	11										
	-	-	9.4	43.1	11.2	50	9.4	23.6		6 [4 - 12]		
Uyttenboogaart 2008	Aspirin	65	46.1	58.4	19.1	23.5	24.7	48.3	14.18 (20.3)	12 [2 - 35]	NA	3 months
	Aspirin and Dipyridamole	22										
	Dipyridamole	1										
	Clopidogrel	1										
	-	-	5.2	38.6	9	36.8	25.5	23.3		13 [2 - 25]		
Couture 2021	Aspirin	62	33.3	74.6	23.9	23.3	23.9	23.3	15.8 (6.2)	16 [11–20]	100	3 months
	Clopidogrel	7										
	Aspirin and Clopidogrel	2										
	-	-	5.2	51.9	10.5	22.6	15.8	22.6		16 [12–20]	100	
Hermann 2009	Aspirin	26	NA	81	47	NA	33	NA	13.6 (22)	10 [1 - 30]	NA	NA
	Aspirin and Clopidogrel	3										
	Aspirin and Dipyridamole	1										
	Clopidogrel	6										
	-	-		69	15		15					
Lin 2021	Aspirin	213	NA	80	44	NA	43.3	27.7	13.8 (7)	NA	NA	3 months
	Clopidogrel	37										
	Aspirin and Clopidogrel	27										
	-	-		74.4	30.6		40	35.9				
Ibrahim 2010	Aspirin	76	23	78	37.3	NA	NA	NA	16 (5.5)	16 [11 - 21]	NA	3 months
	Clopidogrel	15										
	-	-	5	48.9	16.1							
Meurer 2013	NA	NA	27.6	86.9	28.6	NA	31.4	61.1	12.4 (5.7)	NA	NA	at discharge
	-	-	9	62.4	18.8		14.5	31.2				
Chen 2016	NA	NA	30.4	60.9	21.7	30.4	43.5	43.5	10.8 (5.6)	14 [7 - 16]	NA	3 months
	-	-	14.8	60.7	19.7	39.5	33.6	23.8		11 [7 - 14]		
Hang Jing 2020	NA	NA	30.4	87	30.4	8.7	43.5	0	7.8 (6.3)	8 [4 -11]	NA	NA
	-	-	8.3	72.2	25	19.4	11.1	11.1		7 [4 - 13]		
Dorado 2010	Aspirin	55	13.9	69.4	24.3	NA	NA	60.6	13.6 (8.2)	14 [8 - 19]	NA	3 months
	Clopidogrel	14										
	Aspirin and Clopidogrel	2										
	Triflusal	1										
	-	-	25	54				42.3				
Bravo 2008	Aspirin	106	23.4	63.2	29.2	34.1	44.5	39	NA	14	NA	3 months
	Clopidogrel	24										
	Ticlopidine	1										
	Dipyridamole	1										
	Triflusal	5										
	-	-		6	54.3	19.2	30.2	31.4		15		
Tsivgoulis 2018	DAPT	1043	29.7	81.3	30.1	15.2	20.6	57.4	10 (6.5)	9 [6 - 15]	3	3 months
	-	-	26.7	81.4	31	14.9	23.3	59.5		9 [6 - 15]	3.5	
Pan 2015	Aspirin	115	NA	72	26.1	NA	31.9	NA	11.4 (6.7)	12 [8 - 18]	NA	3 months
	Aspirin and Clopidogrel	14										
	-	-		57.4	16.1		15.5			11 [7 - 16]		
Choi 2016	SAPT	353	40.7	77.5	28.4	18.8	44.8	33	10.1 (7.4)	9 [5 - 16]	0	NA
	DAPT	87										
	Triple APT	1										
	-	-	41.4	84	27.2	20.4	46.6	32.7		10 [6 - 15]	0	
Sanak 2012	Aspirin	49	NA	NA	26.8	NA	NA	NA	NA	16	NA	NA
	Clpidogrel	1										
	Aspirin and Dipyridamole	6										
	-	-			15.6					15		
Meseguer 2015	NA	NA	NA	74	16.7	41.9	NA	53.7	11.5 (9.3)	12 [6 - 18]	13.2	3 months
	-	-		41.8	14	37.3		18.8		11 [5 - 18]	15.7	
Diedler 2010	Aspirin	3016	21.9	74.1	20.8	17.9	32.3	51.4	12 (8.9)	NA	NA	3 months
	Clopidogrel	243										
	Aspirin and Dipyridamole	175										
	Aspirin and Clopidogrel	151										
	-	-	4.7	52	13.7	26.9	18.1	26.5				
Peng 2024	SAPT	117670	53,808	114,526	50,631	NA	26,300	82,026	9.85(7.55)	7 [4 -14]	68	3 months
	DAPT	21805										
	-	-	26,221	114,294	41,271		19,171	59,661			9	
											9	

Abbreviations:* IVT* intravenous thrombolysis, *sICH* symptomatic intracranial hemorrhage, *APT* antiplatelet therapy, *SD* standard deviation, *TIA* transient ischemic attack, *HTN* hypertension, *AF* atrial fibrillation, *NIHSS* National Institute of Health Stroke Scale, *IQR* interquartile range, *ENCHANTED* Enhanced Control of Hypertension and Thrombolysis Stroke Study, *RCT* randomized controlled trial, *SITS-MOST* Safe Implementation of Thrombolysis in Stroke-Monitoring Study, *ECASS* European Cooperative Acute Stroke Study, *NINDS* National Institute of Neurological Disorders and Stroke rt-PA Stroke Study Group, *IST* international stroke trial, *mg* milligram, *kg* kilogram, *NA* not available, *SAPT* single antiplatelet therapy, *DAPT* dual antiplatelet therapy

### Quality assessment and publication bias

Four studies had a serious risk of bias, four studies had a low risk of bias, and 22 studies had a moderate risk of bias. The Risk-of-bias plots are presented in (Figure S1). Egger’s test of publication bias was insignificant for all outcomes, sICH (P = 0.1562), any ICH (P = 0.1156), mortality (P = 0.5319), and mRS0-2 (P = 0.069). Funnel plots for all outcomes are presented in (Figures S2-S5).

### Safety outcomes

#### Symptomatic ICH

**Fig. 2:**
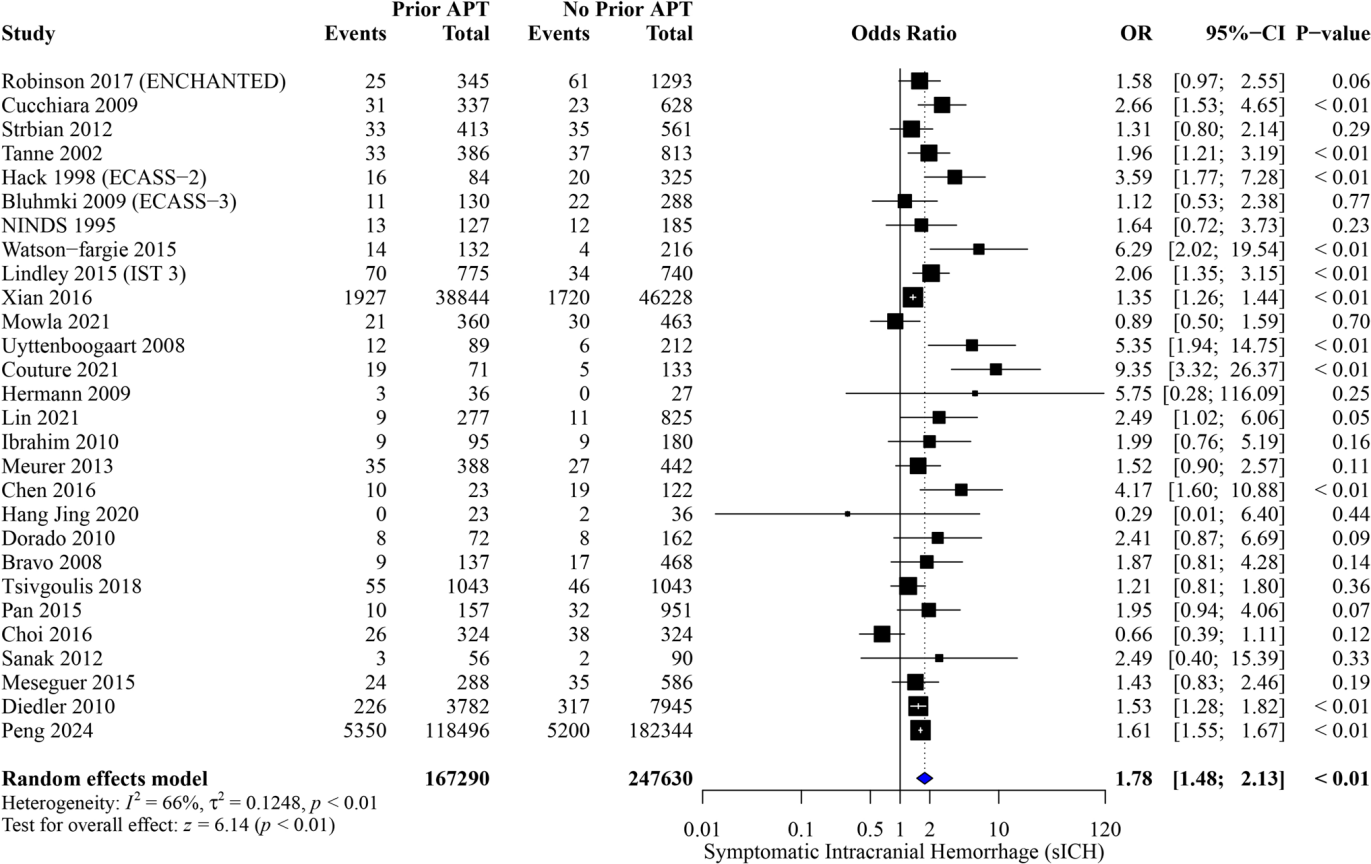
Forest plot of meta-analysis for symptomatic intracranial hemorrhage outcome

Among 414,920 patients, prior APT significantly increased the odds of sICH (OR, 1.78; 95% CI [1.48, 2.13]; *P* < 0.01), with notable heterogeneity between studies (I^2^ = 66%, *P* < 0.01) (Fig. 2). The pooled results were robust through sensitivity analysis, and heterogeneity was best resolved by the exclusion of the study by Xian et al. [16] (I^2^ = 56%) (OR, 1.82; 95% CI [1.50, 2.22]; *P* < 0.01) (Figure S6). The pooled studies analyzed based on different sICH definitions showed consistent associations between prior APT and an increased sICH risk (Figure S7).

Upon subgroup analyses based on study design and baseline NIHSS scores, the association between prior APT and increased sICH risk remains significant. Notably, a baseline NIHSS score of ≥ 10 was associated with a higher risk of sICH among those with APT exposure (OR, 1.96; 95% CI [1.42, 2.70]; *P* < 0.01) than a baseline NIHSS score < 10 (OR, 1.53; 95% CI [1.33, 1.76]; *P* < 0.01) (Figures S8–S9).

In subgroup analysis according to time window from onset to treatment, sICH rates demonstrated a significant increase in risk associated with prior APT use across all time windows. At 3 h, the OR for sICH was 1.73 (95% CI [1.16, 2.57], *P* < 0.01). Similarly, at 4.5 h, the risk remained elevated (OR: 1.37, 95% CI [1.13, 1.67], *P* < 0.01), and the association was even stronger at 6 h (OR: 2.56, 95% CI [1.81, 3.64], *P* < 0.01). A test for subgroup differences across the time windows was significant (P = 0.01), highlighting the differential impact of prior APT on sICH risk depending on the time window. (Figure S10).

#### Any intracranial hemorrhage

**Fig. 3:**
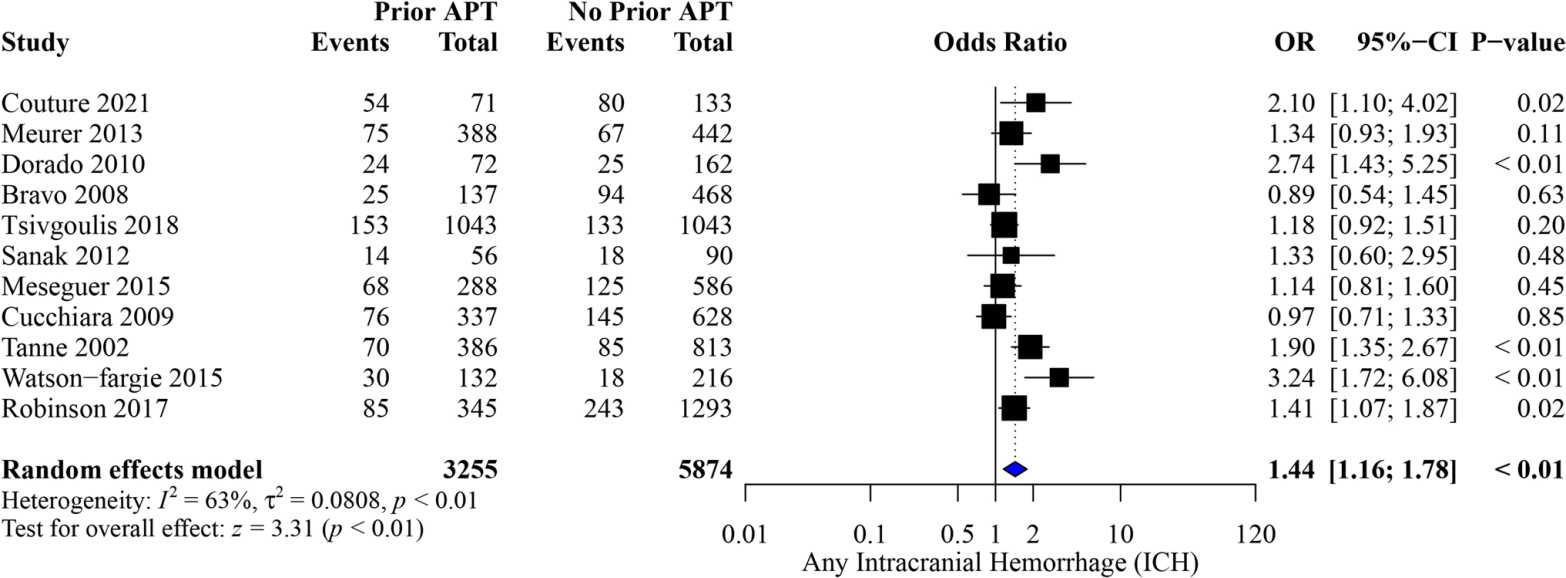
Forest plot of meta-analysis for any intracranial hemorrhage outcome

Among 9,129 patients, prior APT significantly increased the odds of any ICH (OR, 1.44; 95% CI [1.16, 1.78]; *P* < 0.01), with notable heterogeneity (I^2^ = 63%, *P* < 0.01) (Fig. 3). The pooled results were robust through sensitivity analysis, and heterogeneity was best resolved by exclusion of the study by Watson-Fargie et al. [23] (I^2^ = 53%) (OR, 1.34; 95% CI [1.12, 1.61]; *P* < 0.01) (Figure S11). Subgroup analysis based on baseline NIHSS is outlined in (Figure S12).

### Mortality

**Fig. 4:**
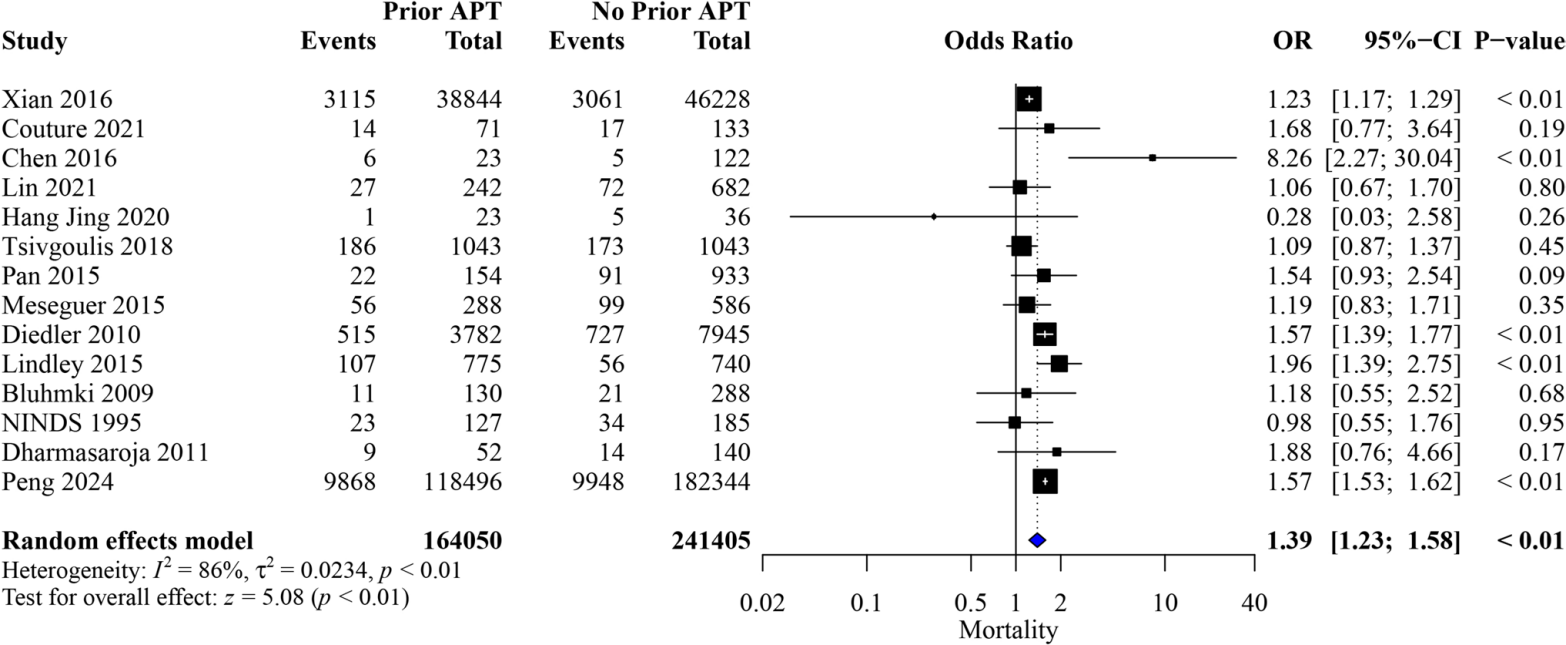
Forest plot of meta-analysis for mortality outcome

Among 405,455 patients, prior APT significantly increased the odds of mortality (OR, 1.39; 95% CI [1.23, 1.58]; *P* < 0.01), with notable heterogeneity (I^2^ = 86%, *P* < 0.01) (Fig. 4). The pooled results were robust through sensitivity analysis, and heterogeneity was best resolved by the exclusion of the study by Xian et al. [16] (I^2^ = 57%) (OR, 1.43; 95% CI [1.24, 1.65]; *P* < 0.01) (Figure S13). Subgroup analyses based on baseline NIHSS score and study design are presented in (Figure S14-S15). Mortality outcomes showed a more complex pattern. At 3 h, the OR was 1.23 (95% CI [0.87, 1.73], P = 0.23). However, at 4.5 h, the odds of mortality were significantly higher for patients with prior APT (OR: 1.41, 95% CI [1.18, 1.69], *P* < 0.01). At the 6-h mark, the OR increased substantially (OR: 3.49, 95% CI [0.88, 13.95], P = 0.08), although this did not reach statistical significance. (Figure S16).

### Efficacy outcomes

#### Good functional outcome

**Fig. 5:**
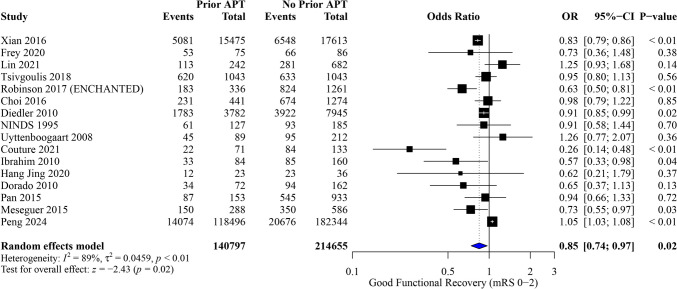
Forest plot of meta-analysis for good functional outcome (mRS 0–2)

Among 355,452 patients, we found that prior APT was associated with lower odds of achieving a good functional outcome (OR, 0.85; 95% CI [0.74, 0.97]; P = 0.02), with notable heterogeneity (I^2^ = 89%, *P* < 0.01) (Fig. 5).

The pooled results were robust through sensitivity analysis, however, the heterogeneity was not resolved (Figure S17). Subgroup analyses based on baseline NIHSS score and study design are presented in (Figures S18 – S17S19). In subgroup analysis according to time window from onset to treatment, no significant differences were observed between the prior APT and no prior APT groups. At 3 h, the OR for good recovery was 0.91 (95% CI [0.72, 1.16], P = 0.46), and at 4.5 h, it was 0.90 (95% CI [0.77, 1.05], P = 0.18). (Figure S20).

#### Poor functional outcome

**Fig. 6:**
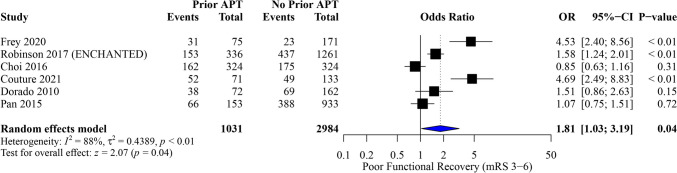
Forest plot of meta-analysis for poor functional outcome (mRS 3–6)

Among 4,015 patients, prior APT significantly increased the odds of poor functional outcome (mRS 3–6) (OR, 1.81; 95% CI [1.03, 3.19]; P = 0.04), with notable heterogeneity (I^2^ = 88%, *P* < 0.01) (Fig. 6). The pooled results were inconsistent through sensitivity analysis, and heterogeneity wasn’t resolved (Figure S21).

## Discussion

Our analysis investigated the safety and efficacy outcomes associated with prior APT in stroke patients treated with IV alteplase. Although there were reviews on using prior APT in AIS cases eligible for IVT, to the authors’ knowledge, this is the largest systematic review and meta-analysis including up-to-data of both subgroup analysis of clinical trials and observational studies and evaluating both efficacy and safety outcome events [10, 24, 25].

In our analysis of 436,232 patients, we found that for AIS patients treated with IV alteplase, pretreatment with APT significantly increased the odds of sICH, any ICH, poor functional outcome, and mortality. Additionally, our subgroup analysis based on time window from onset to treatment showed that prior APT use is associated with an increased risk of sICH, particularly at longer time windows, and may also contribute to higher mortality at 4.5 h, although the evidence for mortality remains inconclusive at 6 h. However, no significant impact of prior APT on good functional recovery was observed across any of the time windows.

Our findings of increased hemorrhagic events and mortality are consistent with the outcomes reported in previous meta-analyses. The observed higher risk of sICH among patients with prior APT was translated into increased poor functional recovery (mRS 3–6) which was not reported in any previous meta-analysis. Furthermore, our analysis revealed a significant association between prior APT and decreased odds of good functional recovery. The existing meta-analyses were heterogeneous regarding the good functional outcomes, with some studies reporting a decreased good functional recovery [14] (OR: 0.86, 95% CI: 0.80—0.93), [26] (OR: 0.69, 95% CI: 0.56—0.85), and [10] (OR: 0.91, 95% CI: 0.88—0.94)], and some other studies reporting no significant association [25] (OR: 0.86, 95% CI: 0.73 – 1.01) and [27] (OR: 0.95, 95% CI: 0.76 – 1.20)] [10, 14, 24, 25, 27].

While the main purpose of thrombolysis therapy is to restore perfusion, up to 10% of patients develop serious adverse effects, of which the sICH is the most feared and can lead to clinical deterioration and poor long-term outcomes [25, 26, 28]. Prior APT, causing inhibition of platelet aggregation, that can continue to the thrombolysis period may be accounted for the higher rates of sICH among prior APT groups [25, 29]. However, due to the lack of experimental and translational research and the complexity of sICH, it might be difficult to elucidate this phenomenon at the mechanistic level fully [14]. A prior study has proposed that recanalization is essential for the occurrence of sICH in the damaged blood vessels beyond the initial blockage sites. This suggests that the increase in sICH might be attributed to reperfusion caused by thrombolysis, which, when combined with platelet inactivation, could elevate the risk of bleeding [30].

Patients using APT often present with a range of comorbidities and risk factors that can influence clinical outcomes. Additionally, the diverse baseline characteristics across populations in the included studies may contribute to the observed effect estimate and partially account for the observed heterogeneity in our analysis. In our subgroup analysis by stroke severity, as measured by the baseline NIHSS, a baseline NIHSS score greater than 10 was associated with higher odds of sICH compared to a baseline NIHSS score of less than 10. This finding suggests that higher baseline stroke severity may amplify the risk of sICH, highlighting the need to consider stroke severity when evaluating the bleeding risks associated with APT in AIS candidates for IV alteplase.

Among the relevant demographic factors, the APT regimen was observed as a significant predictor of hemorrhagic complications after IV alteplase. In the meta-analysis by [27], the highest hemorrhagic risk after IV alteplase among APT users was observed (OR: 2.26, 95CI% [1.39, 3.67], P = 0.001) [27]. This could be explained by the fact that this study included only research studies that investigated prior dual antiplatelet therapy (DAPT). DAPT usually targets more than one pathway for platelet inactivation, hence, increasing the risk of bleeding [16]. This observation warrants a subgroup analysis of the APT regimen and duration; however, we could not do that due to insufficient relevant data. As more antiplatelet agents have become more popular in clinical practice and given the variability in bleeding risk associated with different agents, it is essential to thoroughly evaluate regimens beyond the commonly used regimens aspirin and clopidogrel [31]. Recently, two case reports of successful recanalization using IV alteplase with no bleeding complications in patients on ticagrelor were reported [32]. Therefore, exploring the safety and efficacy of alternative antiplatelet therapies in the context of IV alteplase could provide valuable inputs to better inform clinical decision-making.

Besides the acute timely intervention in AIS cases, good functional outcomes also depend on the sustained patency of cerebral vessels [25, 33]. After the cerebrovascular incident, patients can be at higher risk for platelet aggregation and vascular re-occlusion especially within the first 24 h [34]. Thus, antiplatelet therapy was hypothesized to maintain vascular patency and thus not only sICH but also good functional outcomes. However, previous meta-analyses revealed no significant association between prior APT and recanalization rate with controversial results in improving the functional outcomes [14, 25, 35, 36].

Finally, the existing literature is limited by a lack of evidence regarding the effects of prior APT on outcomes following the use of thrombolytic agents other than alteplase, such as tenecteplase. Considering the unavoidable necessity of APT in patients with cardiovascular and cerebrovascular risk factors, it is imperative to further explore the safety and efficacy profiles of tenecteplase in this specific population.

Our study leveraged an extensive dataset by incorporating the largest population ever analyzed to examine the relationship between prior APT and IV alteplase outcomes. Additionally, by including the most recent studies, we were able to investigate the association between prior APT and poor functional outcomes. To the best of our knowledge, this is the first meta-analysis to report such an association. Furthermore, we performed a comprehensive systematic review and meta-analysis including observational studies that resemble real-world data besides data from subgroup analysis of clinical trials.

The current review does have some limitations. Because of insufficient data, we did not include the outcome of the recanalization rate, and we did not perform subgroup analysis based on the ethnicity of the patients which was proven to affect the outcome of APT administration [10]. Furthermore, there was marked heterogeneity among the studies in certain outcomes although we tried to reduce the heterogeneity using the leave-one-out technique which resolved heterogeneity on some occasions. Although some studies reported agents of prior APT, none provided discrete outcome data for each antiplatelet agent. This limits our ability to fully assess the impact of DAPT on outcomes and highlights a potential area for improvement in future research. The variation in APT doses, regimens, and duration should be considered in future studies. The inherent limitations of observational studies cannot be overcome by meta-analysis as well. Including observational studies, although important to give insights about real-world data, can produce lower-quality evidence. Despite the large included population, almost 93% of them originated from two large-scale studies [16, 17]. The dominance of these two studies may influence the generalizability of our findings and could underrepresent other smaller studies with diverse population. Another important limitation is the utilization of different alteplase doses across the included studies which could impact the outcome results. Insufficient data was the reason behind not being analyzed in our study. Relation between the dose of the IVT agent and hemorrhagic transformation should be assessed in the upcoming studies.

Additionally, A limitation of this study is the incomplete reporting of endovascular thrombectomy utilization across the included studies, with only 13 studies providing this data. This limited our ability to assess the impact of EVT on hemorrhagic transformation and related outcomes. Finally, some included observational studies had an overall high risk of bias which could impact the findings of our study.

### Implications for future research

Despite robust evidence indicating a significant increase in the risk of sICH among patients with prior APT undergoing IVT utilizing alteplase, this relationship needs to be further explored in different APT regimens and other thrombolytics than alteplase. This underscores the need for well-designed research studies with detailed documentation of prior APT duration and regimen. Future research endeavors should also focus on adding adjunctive agents, which might include novel antiplatelets.

## Conclusion

Prior APT was found to significantly increase the risk of sICH, any ICH, mortality, and poor functional outcome, while reducing the likelihood of good functional outcome after IV alteplase. Our findings indicate that specific factors, particularly the severity of stroke and time window from onset to treatment may significantly affect hemorrhagic outcomes in AIS patients undergoing IVT using alteplase. To better understand and mitigate these risks, future research should focus on identifying adjunctive agents that may decrease hemorrhagic complications and investigate the impact of various APT regimens and alternative thrombolytics beyond alteplase on post-thrombolytic outcomes.

## Supplementary Information

Below is the link to the electronic supplementary material.

Supplementary file1 (DOCX 6.14 MB)

## Data Availability

All data generated or analysed during this study are included in this published article [and its supplementary information files].
