# Supplementary material for: Impact of prior antiplatelet therapy on safety and efficacy of alteplase in acute ischemic stroke: a systematic review and meta-analysis
Source: Neurol Sci. 2025 Feb 22;46(6):2461–78. doi: 10.1007/s10072-025-08024-x (PMC12084235; doi:10.1007/s10072-025-08024-x)

**Supplementary Materials**

**Contents:**

Table S1: PRISMA statement 2020 checklist.

Table S2: The detailed search strategy and approach.

Table S3: References of the included studies

Figure S1: Risk of bias graph.

Figure S2: Funnel plot of publication bias for symptomatic intracranial hemorrhage (sICH).

Figure S3: Funnel plot of publication bias for any intracranial hemorrhage (ICH).

Figure S4: Funnel plot of publication bias for mortality.

Figure S5: Funnel plot of publication bias for good functional outcome (mRS 0-2).

Figure S6: Forest plot of sensitivity analysis (Leave-One-Out test) for sICH.

Figure S7: Forest plot of sICH outcome according to different definitions.

Figure S8: Forest plot of subgroup analysis for sICH based on baseline NIHSS.

Figure S9: Forest plot of subgroup analysis for sICH based on study design.

Figure S10: Forest plot of subgroup analysis for sICH based on time window.

Figure S11: Forest plot of sensitivity analysis (Leave-One-Out test) for any ICH outcome.

Figure S12: Forest plot of subgroup analysis for any ICH based on baseline NIHSS.

Figure S13: Forest plot of sensitivity analysis (Leave-One-Out test) for mortality outcome.

Figure S14: Forest plot of subgroup analysis for mortality outcome based on baseline NIHSS.

Figure S15: Forest plot of subgroup analysis for mortality outcome based on study design.

Figure S16: Forest plot of subgroup analysis for mortality outcome based on time window.

Figure S17: Forest plot of sensitivity analysis (Leave-One-Out test) for mRS 0-2 outcome.

Figure S18: Forest plot of subgroup analysis for mRS 0-2 outcome based on baseline NIHSS.

Figure S19: Forest plot of subgroup analysis for mRS 0-2 outcome based on Study design.

Figure S20: Forest plot of subgroup analysis for mRS 0-2 outcome based on time window.

Figure S21: Forest plot of sensitivity analysis (Leave-One-Out test) for mRS 3-6 outcome.

Figure S22: Forest plot of subgroup analysis for mRS 3-6 outcome based on baseline NIHSS.

Figure S23: Forest plot of subgroup analysis for mRS 3-6 outcome based on study design.

Table S1: PRISMA statement 2020 checklist.

| **Section and Topic** | **Item #** | **Checklist item** | **Location where item is reported** |
| --- | --- | --- | --- |
| **TITLE** | | |  |
| Title | 1 | Identify the report as a systematic review. | Page 1 |
| **ABSTRACT** | | |  |
| Abstract | 2 | See the PRISMA 2020 for Abstracts checklist. | Page 2 |
| **INTRODUCTION** | | |  |
| Rationale | 3 | Describe the rationale for the review in the context of existing knowledge. | Page 3 |
| Objectives | 4 | Provide an explicit statement of the objective(s) or question(s) the review addresses. | Page 3 |
| **METHODS** | | |  |
| Eligibility criteria | 5 | Specify the inclusion and exclusion criteria for the review and how studies were grouped for the syntheses. | Page 4 |
| Information sources | 6 | Specify all databases, registers, websites, organisations, reference lists and other sources searched or consulted to identify studies. Specify the date when each source was last searched or consulted. | Pages 3, 4 |
| Search strategy | 7 | Present the full search strategies for all databases, registers and websites, including any filters and limits used. | Supplement: Table S2 |
| Selection process | 8 | Specify the methods used to decide whether a study met the inclusion criteria of the review, including how many reviewers screened each record and each report retrieved, whether they worked independently, and if applicable, details of automation tools used in the process. | Page 4 |
| Data collection process | 9 | Specify the methods used to collect data from reports, including how many reviewers collected data from each report, whether they worked independently, any processes for obtaining or confirming data from study investigators, and if applicable, details of automation tools used in the process. | Page 4 |
| Data items | 10a | List and define all outcomes for which data were sought. Specify whether all results that were compatible with each outcome domain in each study were sought (e.g. for all measures, time points, analyses), and if not, the methods used to decide which results to collect. | Page 4 |
|  | 10b | List and define all other variables for which data were sought (e.g. participant and intervention characteristics, funding sources). Describe any assumptions made about any missing or unclear information. | Page 4 |
| Study risk of bias assessment | 11 | Specify the methods used to assess risk of bias in the included studies, including details of the tool(s) used, how many reviewers assessed each study and whether they worked independently, and if applicable, details of automation tools used in the process. | Page 5 |
| Effect measures | 12 | Specify for each outcome the effect measure(s) (e.g. risk ratio, mean difference) used in the synthesis or presentation of results. | Page 5 |
| Synthesis methods | 13a | Describe the processes used to decide which studies were eligible for each synthesis (e.g. tabulating the study intervention characteristics and comparing against the planned groups for each synthesis (item #5)). | Page 4 |
|  | 13b | Describe any methods required to prepare the data for presentation or synthesis, such as handling of missing summary statistics, or data conversions. | Page 5 |
|  | 13c | Describe any methods used to tabulate or visually display results of individual studies and syntheses. | Page 5 |
|  | 13d | Describe any methods used to synthesize results and provide a rationale for the choice(s). If meta-analysis was performed, describe the model(s), method(s) to identify the presence and extent of statistical heterogeneity, and software package(s) used. | Page 5 |
|  | 13e | Describe any methods used to explore possible causes of heterogeneity among study results (e.g. subgroup analysis, meta-regression). | Page 5 |
|  | 13f | Describe any sensitivity analyses conducted to assess robustness of the synthesized results. | Page 5 |
| Reporting bias assessment | 14 | Describe any methods used to assess risk of bias due to missing results in a synthesis (arising from reporting biases). | Page 5 |
| Certainty assessment | 15 | Describe any methods used to assess certainty (or confidence) in the body of evidence for an outcome. | Page 5 |
| **RESULTS** | | |  |
| Study selection | 16a | Describe the results of the search and selection process, from the number of records identified in the search to the number of studies included in the review, ideally using a flow diagram. | Page 5 |
|  | 16b | Cite studies that might appear to meet the inclusion criteria, but which were excluded, and explain why they were excluded. | Not applicable |
| Study characteristics | 17 | Cite each included study and present its characteristics. | Page 5 |
| Risk of bias in studies | 18 | Present assessments of risk of bias for each included study. | Page 5 |
| Results of individual studies | 19 | For all outcomes, present, for each study: (a) summary statistics for each group (where appropriate) and (b) an effect estimate and its precision (e.g. confidence/credible interval), ideally using structured tables or plots. | Pages 5, 6 |
| Results of syntheses | 20a | For each synthesis, briefly summarise the characteristics and risk of bias among contributing studies. | Pages 5, 6 |
|  | 20b | Present results of all statistical syntheses conducted. If meta-analysis was done, present for each the summary estimate and its precision (e.g. confidence/credible interval) and measures of statistical heterogeneity. If comparing groups, describe the direction of the effect. | Pages 5, 6 |
|  | 20c | Present results of all investigations of possible causes of heterogeneity among study results. | Pages 5, 6 |
|  | 20d | Present results of all sensitivity analyses conducted to assess the robustness of the synthesized results. | Pages 5, 6 |
| Reporting biases | 21 | Present assessments of risk of bias due to missing results (arising from reporting biases) for each synthesis assessed. | Not applicable |
| Certainty of evidence | 22 | Present assessments of certainty (or confidence) in the body of evidence for each outcome assessed. | Supplement: Table S3 |
| **DISCUSSION** | | |  |
| Discussion | 23a | Provide a general interpretation of the results in the context of other evidence. | Page 7 |
|  | 23b | Discuss any limitations of the evidence included in the review. | Page 8 |
|  | 23c | Discuss any limitations of the review processes used. | Page 8 |
|  | 23d | Discuss implications of the results for practice, policy, and future research. | Pages 8, 9 |
| **OTHER INFORMATION** | | |  |
| Registration and protocol | 24a | Provide registration information for the review, including register name and registration number, or state that the review was not registered. | Page 3 |
|  | 24b | Indicate where the review protocol can be accessed, or state that a protocol was not prepared. | Page 3 |
|  | 24c | Describe and explain any amendments to information provided at registration or in the protocol. | Not applicable |
| Support | 25 | Describe sources of financial or non-financial support for the review, and the role of the funders or sponsors in the review. | Page 9 |
| Competing interests | 26 | Declare any competing interests of review authors. | Page 9 |
| Availability of data, code and other materials | 27 | Report which of the following are publicly available and where they can be found: template data collection forms; data extracted from included studies; data used for all analyses; analytic code; any other materials used in the review. | Page 9 |

Table S2: The detailed search strategy and approach.

| Author | Database | Search strategy | Number of results | Date |
| --- | --- | --- | --- | --- |
| Ahmed Naeem | Pubmed | ((stroke OR "ischemic stroke" OR "cerebral infarction" OR "cerebral ischemia" OR " brain ischemia" OR " brain infarction") AND (“IV thrombolysis” OR “Intravenous thrombolysis” OR “Intra-venous thrombolysis” OR “tissue plasminogen activator” OR tpa OR alteplase) AND (antiplatelet OR anti-platelet OR "platelet inhibitors" OR DAPT OR ASA OR aspirin OR “dual antiplatelet” OR clopidogrel OR ticagrelor OR prasugrel) AND (prior OR "prior use" OR preceding OR before OR premedication OR pre-medication OR pretreatment OR pre-treatment)) | 131 | 30/06/2024 |
| Ahmed Naeem | Cochrane | ((stroke OR "ischemic stroke" OR "cerebral infarction" OR "cerebral ischemia" OR " brain ischemia" OR " brain infarction") AND (“IV thrombolysis” OR “Intravenous thrombolysis” OR “Intra-venous thrombolysis” OR “tissue plasminogen activator” OR tpa OR alteplase) AND (antiplatelet OR anti-platelet OR "platelet inhibitors" OR DAPT OR ASA OR aspirin OR “dual antiplatelet” OR clopidogrel OR ticagrelor OR prasugrel) AND (prior OR "prior use" OR preceding OR before OR premedication OR pre-medication OR pretreatment OR pre-treatment)) | 72 | 30/06/2024 |
| Ahmed Naeem | WOS | ((stroke OR "ischemic stroke" OR "cerebral infarction" OR "cerebral ischemia" OR " brain ischemia" OR " brain infarction") AND (“IV thrombolysis” OR “Intravenous thrombolysis” OR “Intra-venous thrombolysis” OR “tissue plasminogen activator” OR tpa OR alteplase) AND (antiplatelet OR anti-platelet OR "platelet inhibitors" OR DAPT OR ASA OR aspirin OR “dual antiplatelet” OR clopidogrel OR ticagrelor OR prasugrel) AND (prior OR "prior use" OR preceding OR before OR premedication OR pre-medication OR pretreatment OR pre-treatment)) | 234 | 30/06/2024 |
| Ahmed Naeem | Scopus | ((stroke OR "ischemic stroke" OR "cerebral infarction" OR "cerebral ischemia" OR " brain ischemia" OR " brain infarction") AND (“IV thrombolysis” OR “Intravenous thrombolysis” OR “Intra-venous thrombolysis” OR “tissue plasminogen activator” OR tpa OR alteplase) AND (antiplatelet OR anti-platelet OR "platelet inhibitors" OR DAPT OR ASA OR aspirin OR “dual antiplatelet” OR clopidogrel OR ticagrelor OR prasugrel) AND (prior OR "prior use" OR preceding OR before OR premedication OR pre-medication OR pretreatment OR pre-treatment)) | 268 | 30/06/2024 |

| Table S3. References of the included studies | |
| --- | --- |
| Study ID | Reference |
| Bluhmki 2009 (ECASS-3) | Bluhmki E, Chamorro Á, Dávalos A, et al (2009) Stroke treatment with alteplase given 3·0-4·5 h after onset of acute ischaemic stroke (ECASS III): additional outcomes and subgroup analysis of a randomised controlled trial. Lancet Neurol 8:1095–1102. https://doi.org/10.1016/S1474-4422(09)70264-9 |
| Bravo 2008 | Bravo Y, Martí-Fàbregas J, Cocho D, et al (2008) Influence of antiplatelet pre-treatment on the risk of symptomatic intracranial haemorrhage after intravenous thrombolysis. Cerebrovasc Dis 26:126–133. https://doi.org/10.1159/000139659 |
| Chen 2016 | Chen S, Lu X, Zhang W, et al (2016) Does prior antiplatelet treatment increase the risk of hemorrhagic transformation and unfavorable outcome on day 90 after intravenous thrombolysis in acute ischemic stroke patients? J Stroke Cerebrovasc Dis 25:1366–1370. https://doi.org/10.1016/j.jstrokecerebrovasdis.2016.01.038 |
| Choi 2016 | Choi JC, Lee JS, Park TH, et al (2016) Prestroke antiplatelet effect on symptomatic intracranial hemorrhage and functional outcome in intravenous thrombolysis. J Stroke 18:344–351. https://doi.org/10.5853/jos.2016.00185 |
| Couture 2021 | Couture M, Marnat G, Griffier R, et al (2021) Antiplatelet therapy increases symptomatic ICH risk after thrombolysis and thrombectomy. Acta Neurol Scand 144:500–508. https://doi.org/10.1111/ane.13468 |
| Cucchiara 2009 | Cucchiara B, Kasner SE, Tanne D, et al (2009) Factors associated with intracerebral hemorrhage after thrombolytic therapy for ischemic stroke: pooled analysis of placebo data from the Stroke-Acute Ischemic NXY Treatment (SAINT) I and SAINT II Trials. Stroke 40:3067–3072. https://doi.org/10.1161/STROKEAHA.109.554386 |
| Dharmasaroja 2011 | Dharmasaroja PA, Dharmasaroja P, Muengtaweepongsa S (2011) Outcomes of Thai patients with acute ischemic stroke after intravenous thrombolysis. J Neurol Sci 300:74–77. https://doi.org/10.1016/j.jns.2010.09.029 |
| Diedler 2010 | Diedler J, Ahmed N, Sykora M, et al (2010) Safety of intravenous thrombolysis for acute ischemic stroke in patients receiving antiplatelet therapy at stroke onset. Stroke 41:288–294. https://doi.org/10.1161/STROKEAHA.109.559724 |
| Dorado 2010 | Dorado L, Millán M, De La Ossa NP, et al (2010) Influence of antiplatelet pre-treatment on the risk of intracranial haemorrhage in acute ischaemic stroke after intravenous thrombolysis. Eur J Neurol 17:301–306. https://doi.org/10.1111/j.1468-1331.2009.02843.x |
| Frey 2020 | Frey BM, Boutitie F, Cheng B, et al (2020) Safety and efficacy of intravenous thrombolysis in stroke patients on prior antiplatelet therapy in the WAKE-UP trial. Neurol Res Pract 2:40. https://doi.org/10.1186/s42466-020-00087-9 |
| Hack 1998 (ECASS-2) | Hacke W, Kaste M, Fieschi C, et al (1998) Randomised double-blind placebo-controlled trial of thrombolytic therapy with intravenous alteplase in acute ischaemic stroke (ECASS II). Lancet 352:1245–1251. https://doi.org/10.1016/S0140-6736(98)08020-9 |
| Hermann 2009 | Hermann A, Dzialowski I, Koch R, Gahn G (2009) Combined anti-platelet therapy with aspirin and clopidogrel: Risk factor for thrombolysis-related intracerebral hemorrhage in acute ischemic stroke? J Neurol Sci 284:155–157. https://doi.org/10.1016/j.jns.2009.05.003 |
| Ibrahim 2010 | Ibrahim MM, Sebastian J, Hussain M, et al (2010) Does current oral antiplatelet agent or subtherapeutic anticoagulation use have an effect on tissue-plasminogen-activator-mediated recanalization rate in patients with acute ischemic stroke? Cerebrovasc Dis 30:508–513. https://doi.org/10.1159/000319029 |
| Hang Jing 2020 | Jing H, Yi-Jia G, Bai-Li S, et al (2020) Effect of antiplatelet pretreatment on platelet aggregation and clinical outcomes in acute ischemic stroke patients treated with recombinant tissue-type plasminogen activator. Pharmazie 75:23–26. https://doi.org/10.1691/ph.2020.9826 |
| Lin 2021 | Lin S-F, Hu H-H, Ho B-L, et al (2021) Pre-treatment of Single and Double Antiplatelet and Anticoagulant With Intravenous Thrombolysis for Older Adults With Acute Ischemic Stroke: The TTT-AIS Experience. Front Neurol 12:. https://doi.org/10.3389/fneur.2021.628077 |
| Lindley 2015 (IST 3) | Lindley RI, Wardlaw JM, Whiteley WN, et al (2015) Alteplase for acute ischemic stroke: Outcomes by clinically important subgroups in the Third International Stroke Trial. Stroke 46:746–756. https://doi.org/10.1161/STROKEAHA.114.006573 |
| Meseguer 2015 | Meseguer E, Labreuche J, Guidoux C, et al (2015) Outcomes after stroke thrombolysis according to prior antiplatelet use. Int J Stroke 10:163–169. https://doi.org/10.1111/ijs.12421 |
| Meurer 2013 | Meurer WJ, Kwok H, Skolarus LE, et al (2013) Does preexisting antiplatelet treatment influence postthrombolysis intracranial hemorrhage in community-treated ischemic stroke patients? An observational study. Acad Emerg Med 20:146–154. https://doi.org/10.1111/acem.12077 |
| Mowla 2021 | Mowla A, Sharifian-Dorche M, Mehla S, et al (2021) Safety and efficacy of antiplatelet use before intravenous thrombolysis for acute Ischemic stroke. J Neurol Sci 425:117451. https://doi.org/10.1016/j.jns.2021.117451 |
| NINDS 1995 | National Institute of Neurological Disorders and Stroke rt-PA Stroke Study Group (1995) Tissue Plasminogen Activator for Acute Ischemic Stroke. N Engl J Med 333:1581–1588. https://doi.org/10.1056/NEJM199512143332401 |
| Pan 2015 | Pan Y, Chen Q, Liao X, et al (2015) Preexisting dual antiplatelet treatment increases the risk of post-thrombolysis intracranial hemorrhage in Chinese stroke patients. Neurol Res 37:64–68. https://doi.org/10.1179/1743132814Y.0000000390 |
| Peng 2024 | Peng TJ, Schwamm LH, Fonarow GC, et al (2024) Contemporary Prestroke Dual Antiplatelet Use and Symptomatic Intracerebral Hemorrhage Risk After Thrombolysis. JAMA Neurol 81:722. https://doi.org/10.1001/jamaneurol.2024.1312 |
| Robinson 2017 (ENCHANTED) | Robinson TG, Wang X, Arima H, et al (2017) Low-Versus Standard-Dose Alteplase in Patients on Prior Antiplatelet Therapy the ENCHANTED Trial (Enhanced Control of Hypertension and Thrombolysis Stroke Study). Stroke 48:1877–1883. https://doi.org/10.1161/STROKEAHA.116.016274 |
| Sanak 2012 | Šaňák D, Kuliha M, Herzig R, et al (2012) Prior use of antiplatelet therapy can be associated with a higher chance for early recanalization of the occluded middle cerebral artery in acute stroke patients treated with intravenous thrombolysis. Eur Neurol 67:52–56. https://doi.org/10.1159/000333064 |
| Strbian 2012 | Strbian D, Engelter S, Michel P, et al (2012) Symptomatic intracranial hemorrhage after stroke thrombolysis: The SEDAN score. Ann Neurol 71:634–641. https://doi.org/10.1002/ana.23546 |
| Tanne 2002 | Tanne D, Kasner SE, Demchuk AM, et al (2002) Markers of increased risk of intracerebral hemorrhage after intravenous recombinant tissue plasminogen activator therapy for acute ischemic stroke in clinical practice: The multicenter rt-PA acute stroke survey. Circulation 105:1679–1685. https://doi.org/10.1161/01.CIR.0000012747.53592.6A |
| Tsivgoulis 2018 | Tsivgoulis G, Goyal N, Kerro A, et al (2018) Dual antiplatelet therapy pretreatment in IV thrombolysis for acute ischemic stroke. Neurology 91:. https://doi.org/10.1212/WNL.0000000000006168 |
| Uyttenboogaart 2008 | Uyttenboogaart M, Koch MW, Koopman K, et al (2008) Safety of antiplatelet therapy prior to intravenous thrombolysis in acute ischemic stroke. Arch Neurol 65:607–611. https://doi.org/10.1001/archneur.65.5.noc70077 |
| Watson-fargie 2015 | Watson-Fargie T, Dai D, MacLeod MJ, Reid JM (2015) Comparison of predictive scores of symptomatic intracerebral haemorrhage after stroke thrombolysis in a single centre. J R Coll Physicians Edinb 45:127–132. https://doi.org/10.4997/JRCPE.2015.208 |
| Xian 2016 | Xian Y, Federspiel JJ, Grau-Sepulveda M, et al (2016) Risks and benefits associated with prestroke antiplatelet therapy among patients with acute ischemic stroke treated with intravenous tissue plasminogen activator. JAMA Neurol 73:50–59. https://doi.org/10.1001/jamaneurol.2015.3106 |

Figure S1: Risk of bias graph. The panel presents a schematic representation of risks (low = green, moderate = yellow, and serious = red) for specific types of biases of each study in the review.
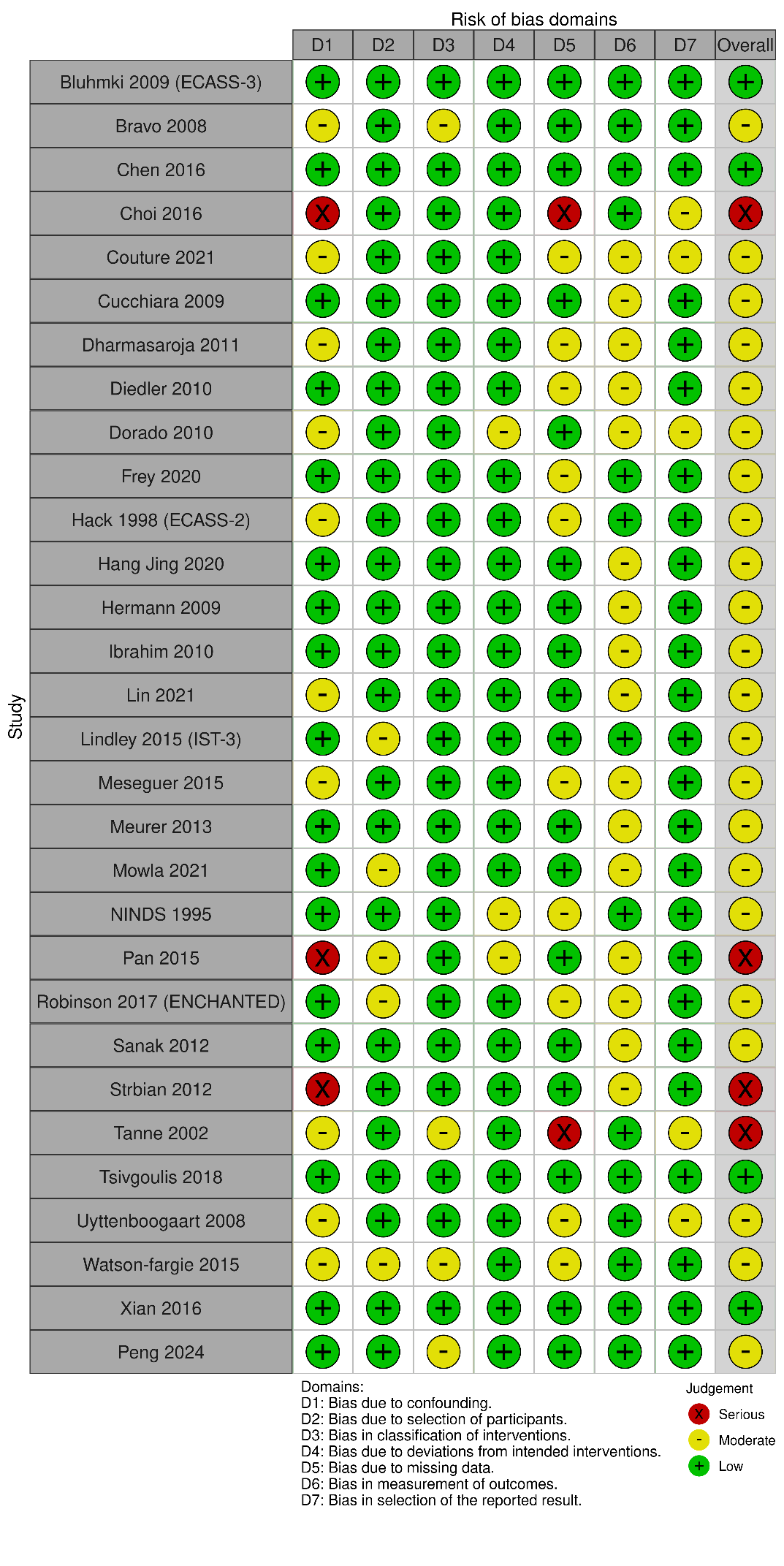


Figure S2: Funnel plot of publication bias for symptomatic intracranial hemorrhage (sICH) outcome.


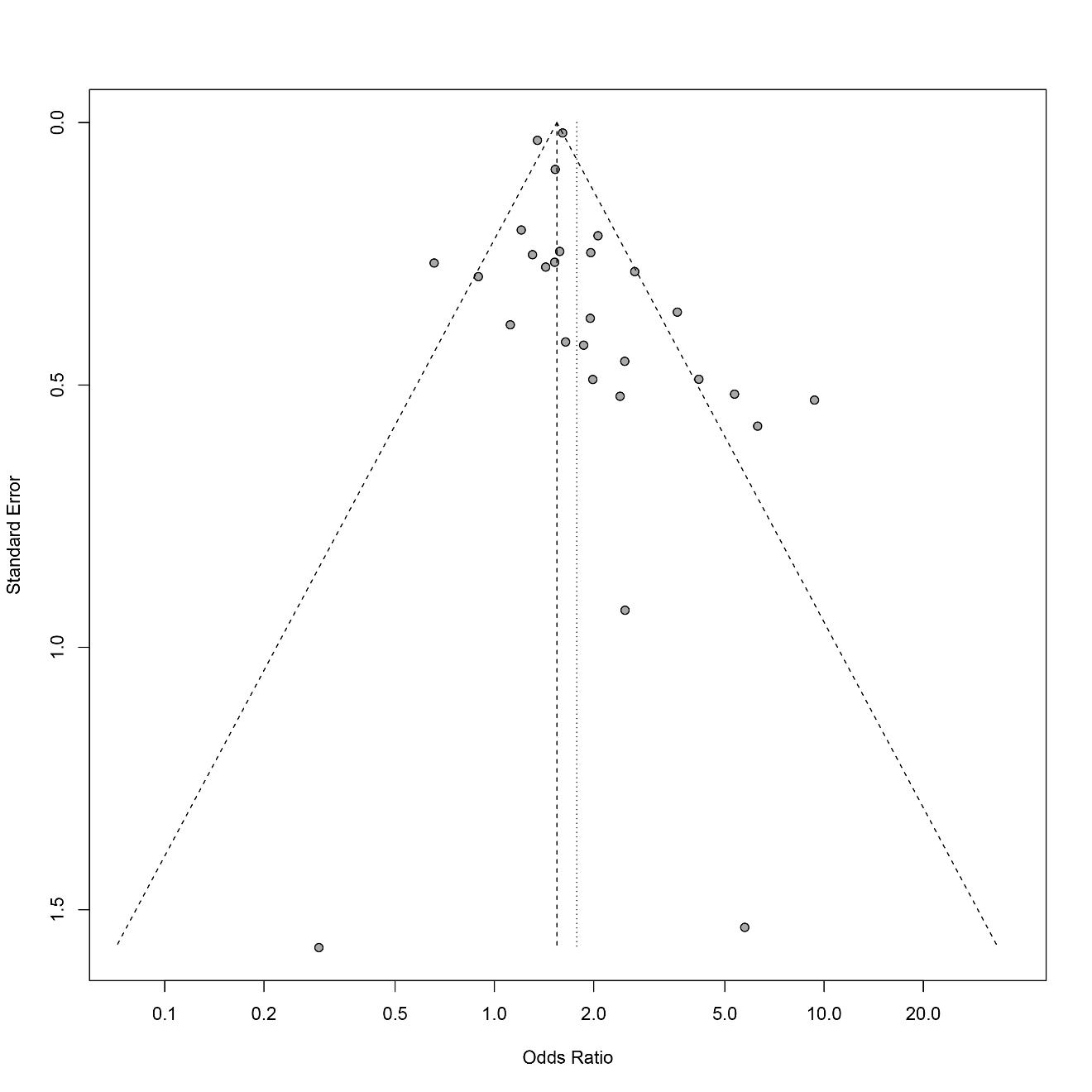


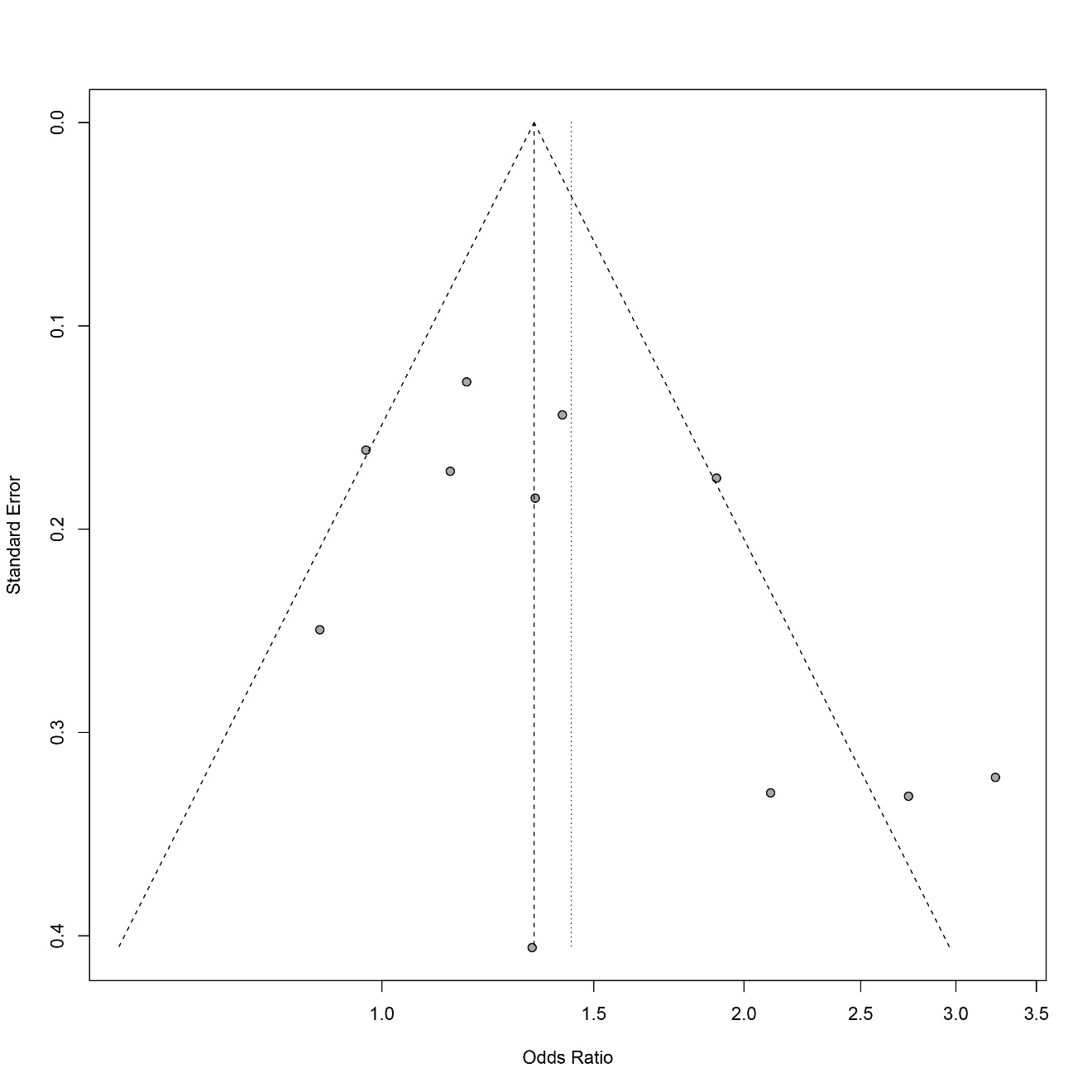
Figure S3: Funnel plot of publication bias for any intracranial hemorrhage (ICH) outcome.

Figure S4: Funnel plot of publication bias for mortality outcome.


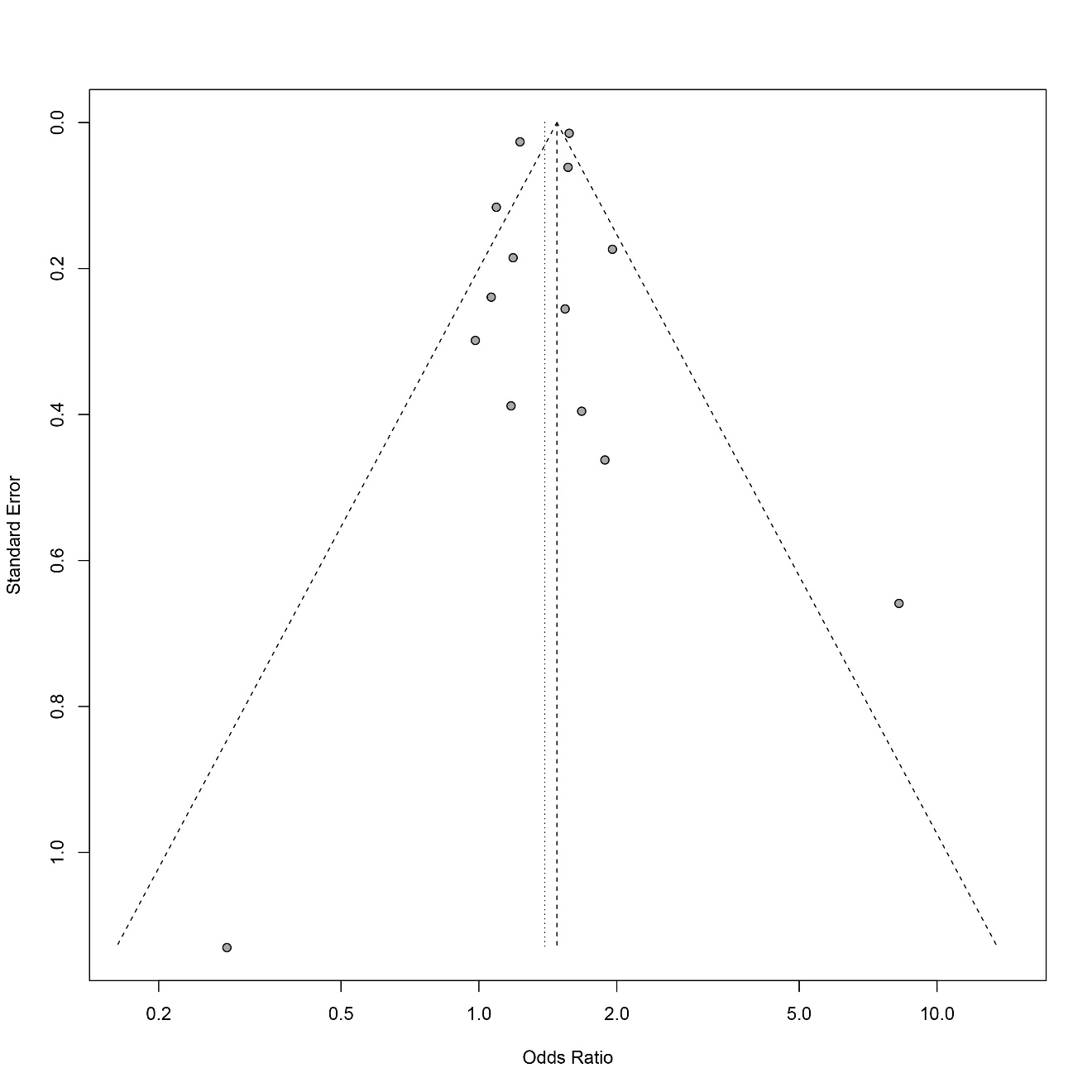


Figure S5: Funnel plot of publication bias for good functional outcome (mRS 0-2).


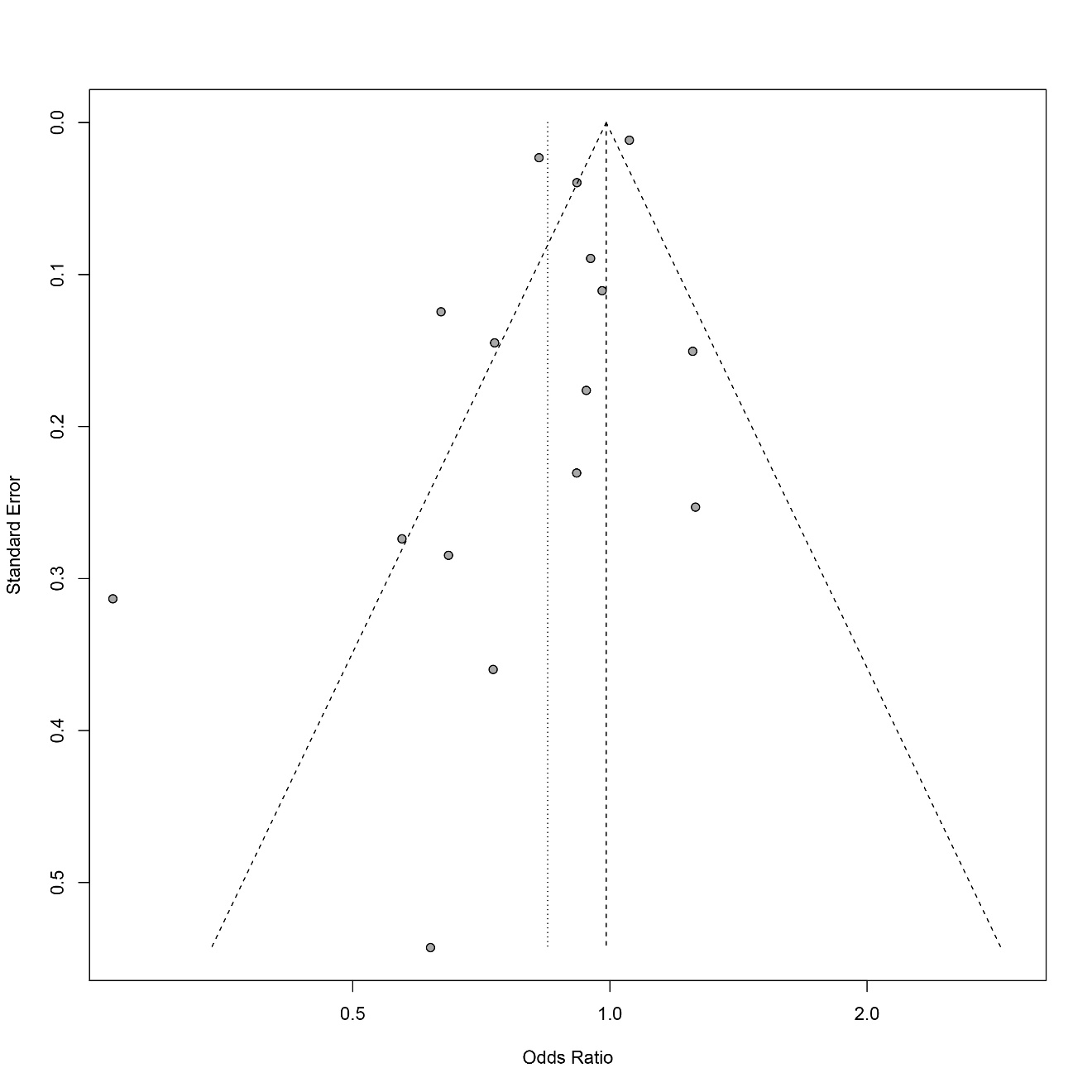


Figure S6: Forest plot of sensitivity analysis (Leave-One-Out test) for sICH outcome.


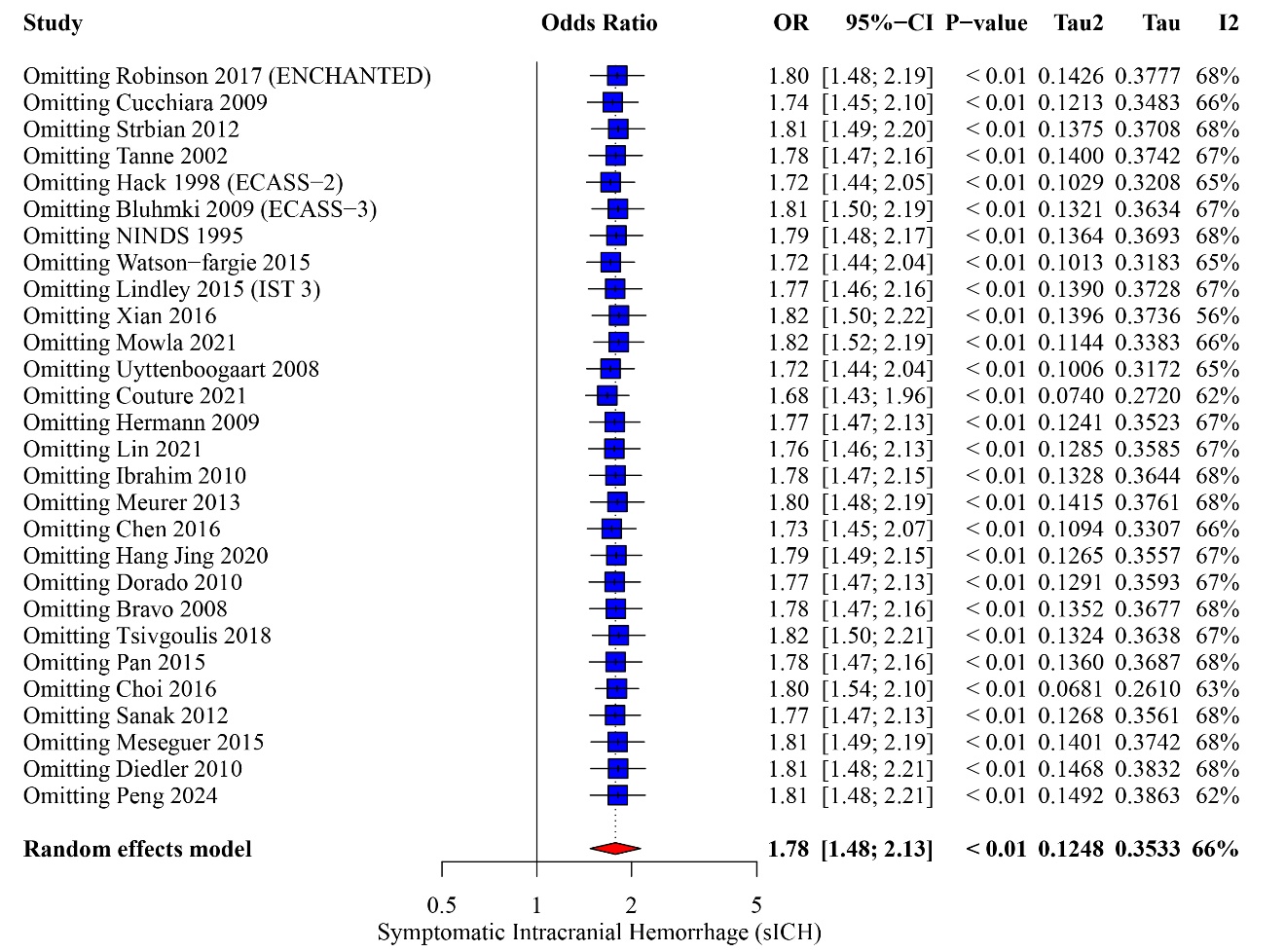


Figure S7: Forest plot of sICH outcome according to different definitions.


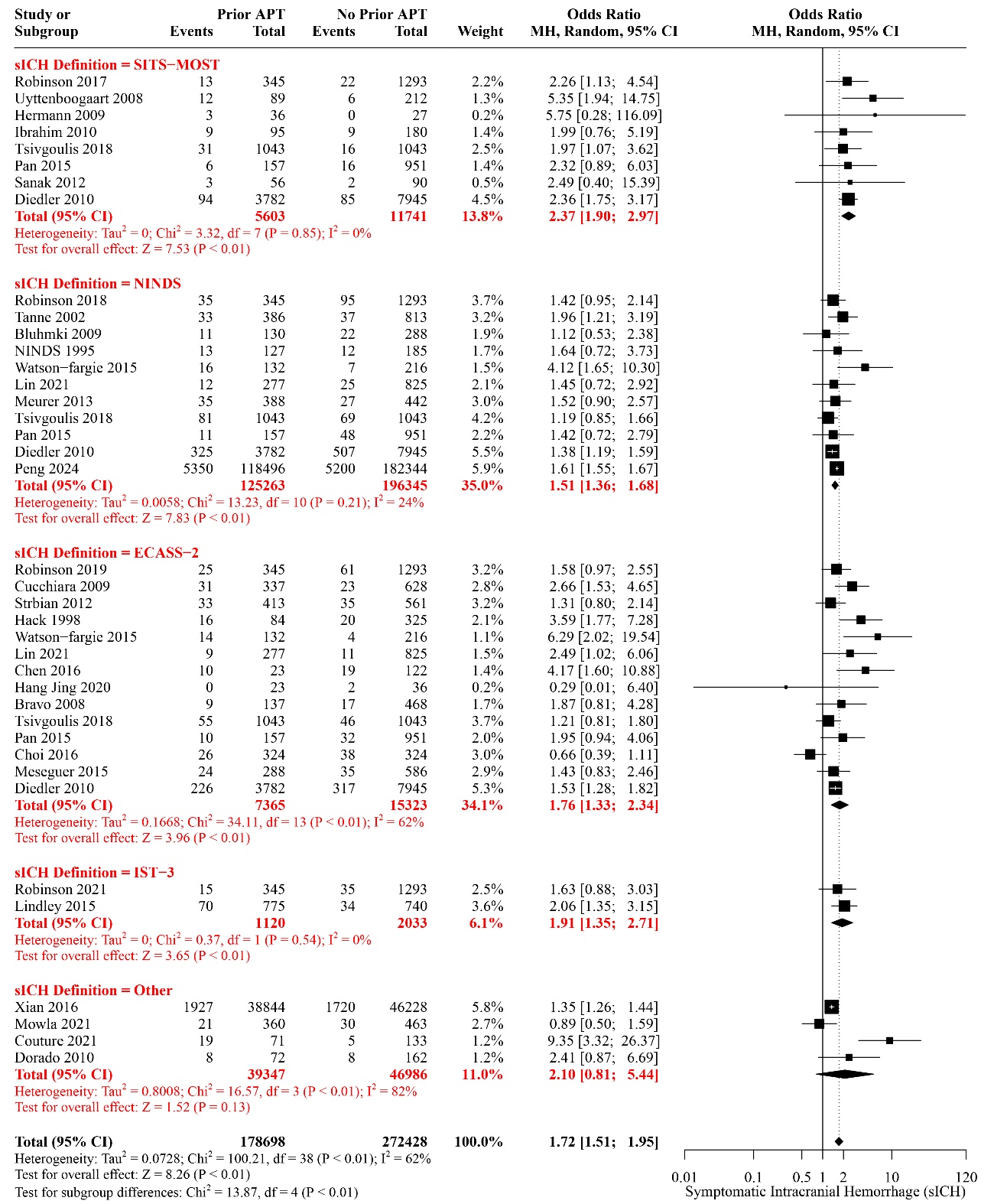


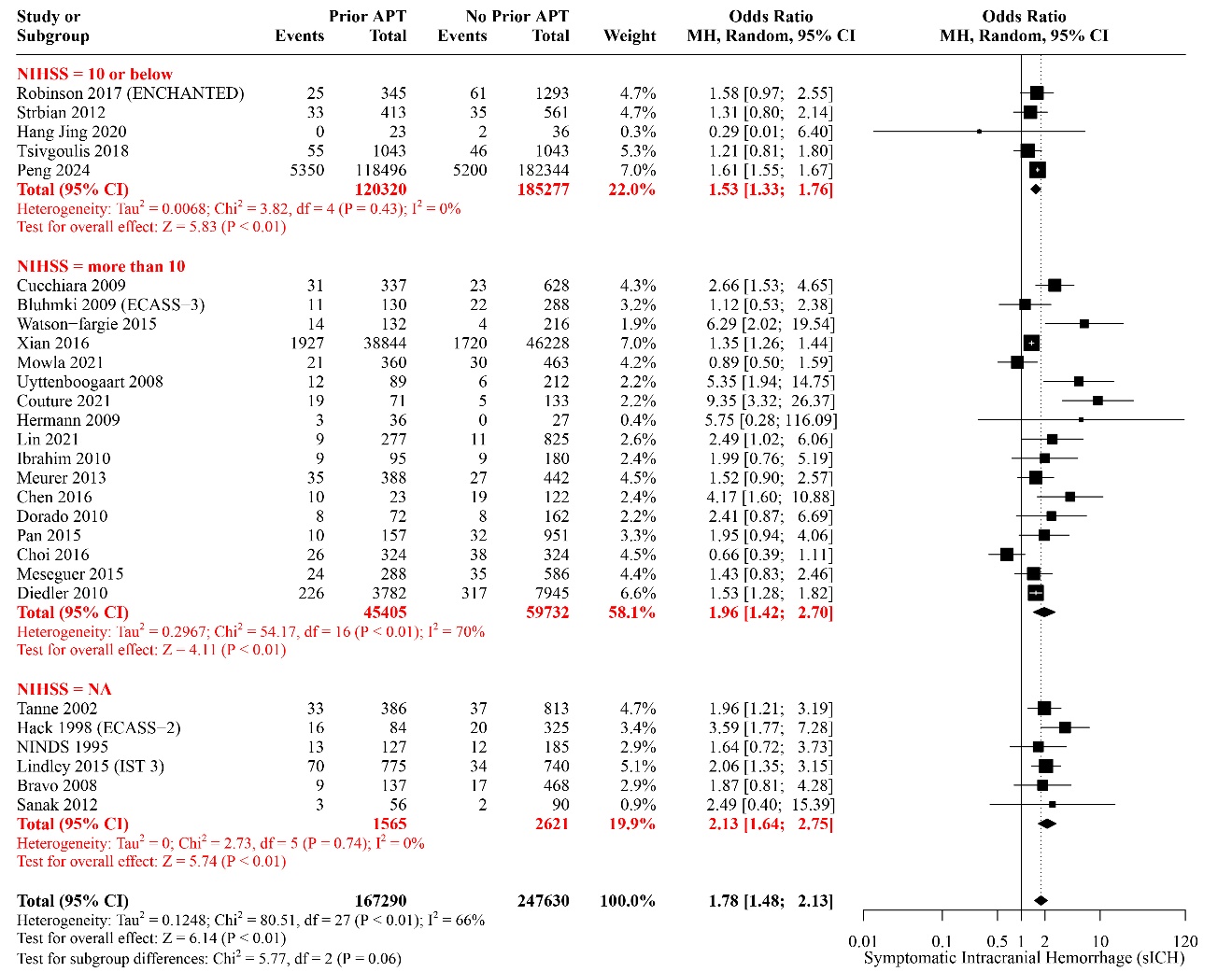
Figure S8: Forest plot of subgroup analysis for sICH based on baseline NIHSS.

Figure S9: Forest plot of subgroup analysis for sICH based on study design.


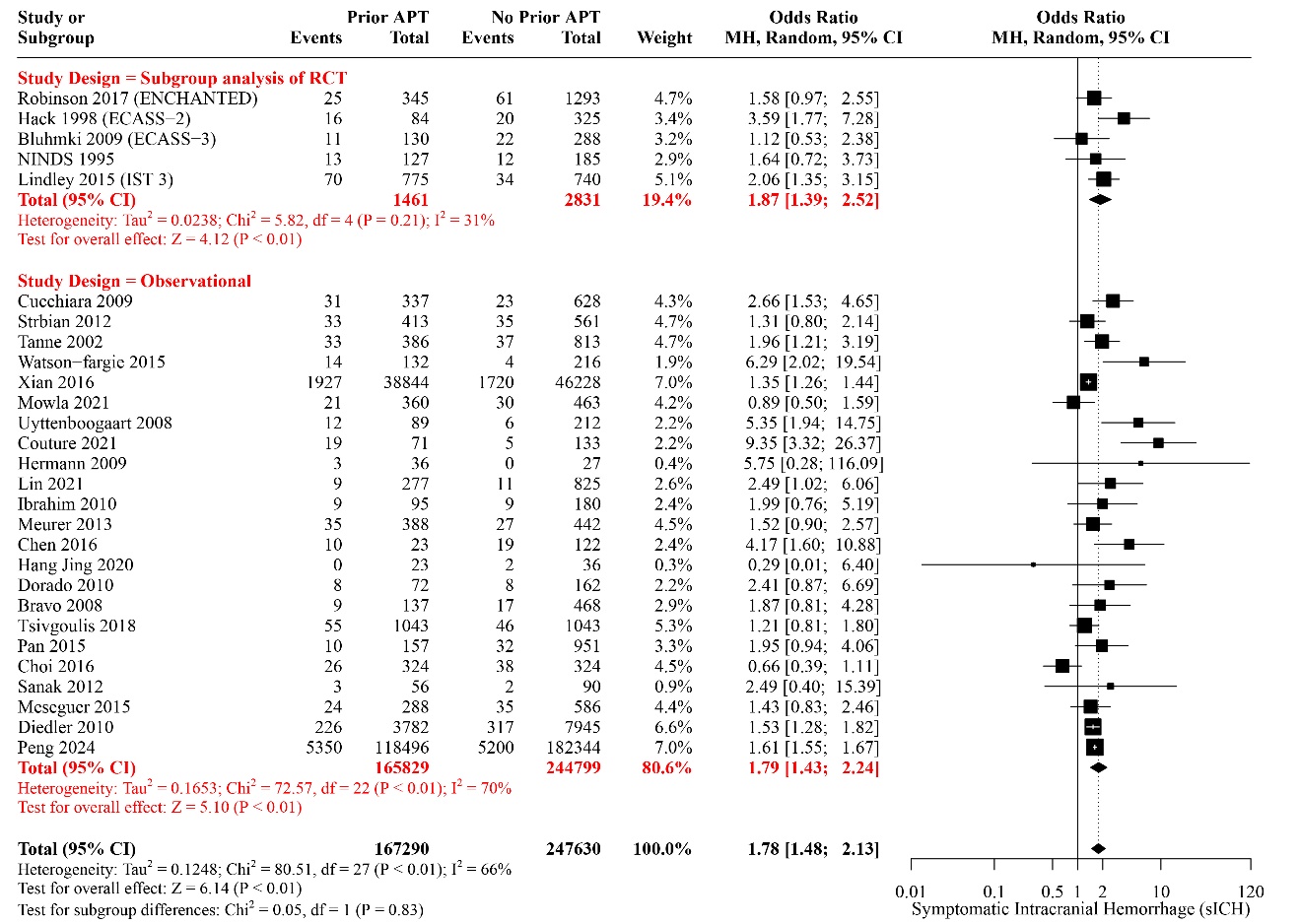

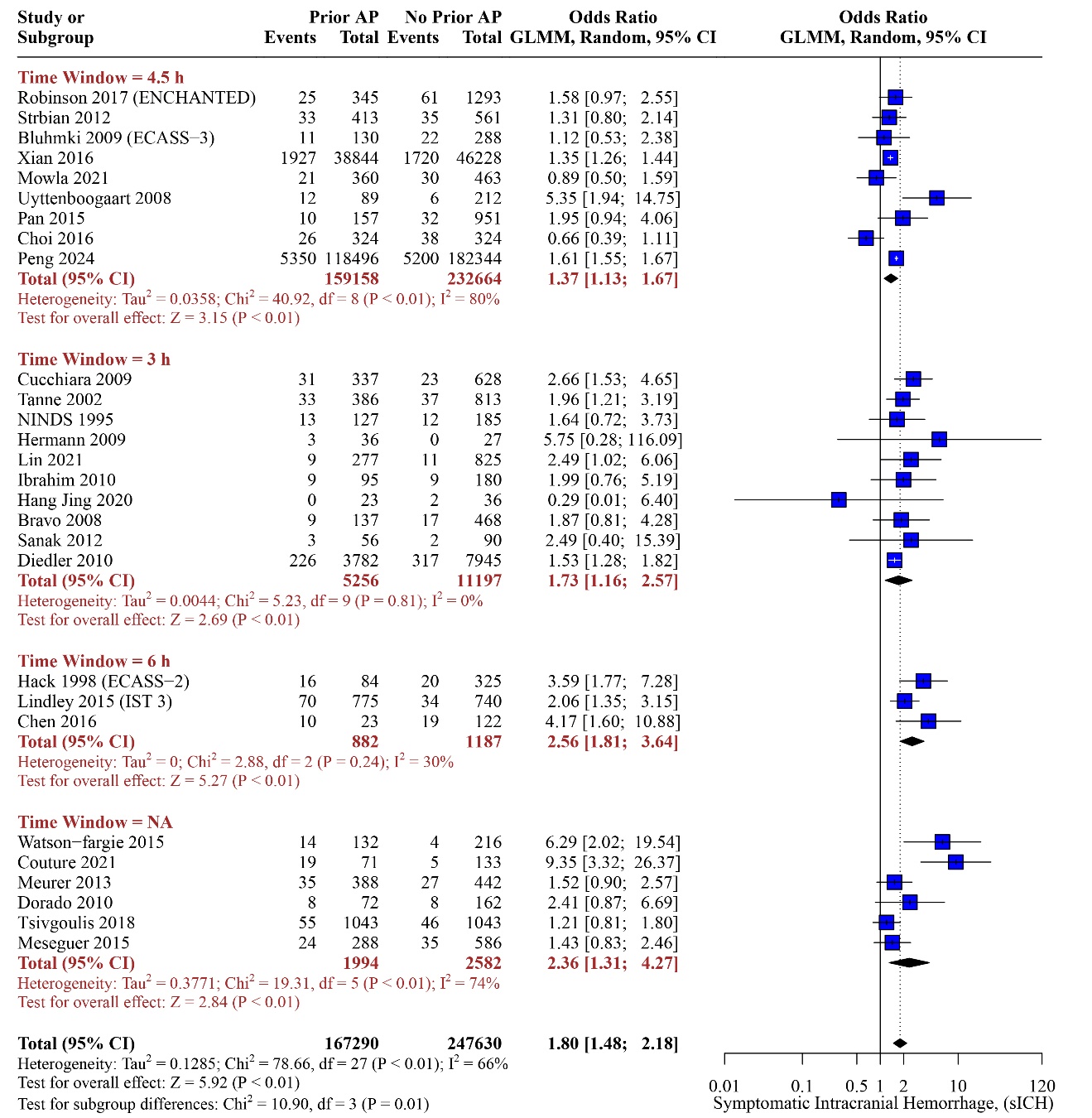
Figure S10: Forest plot for subgroup analysis of sICH outcome based on time window.

Figure S11: Forest plot of sensitivity analysis (Leave-One-Out test) for any ICH outcome.


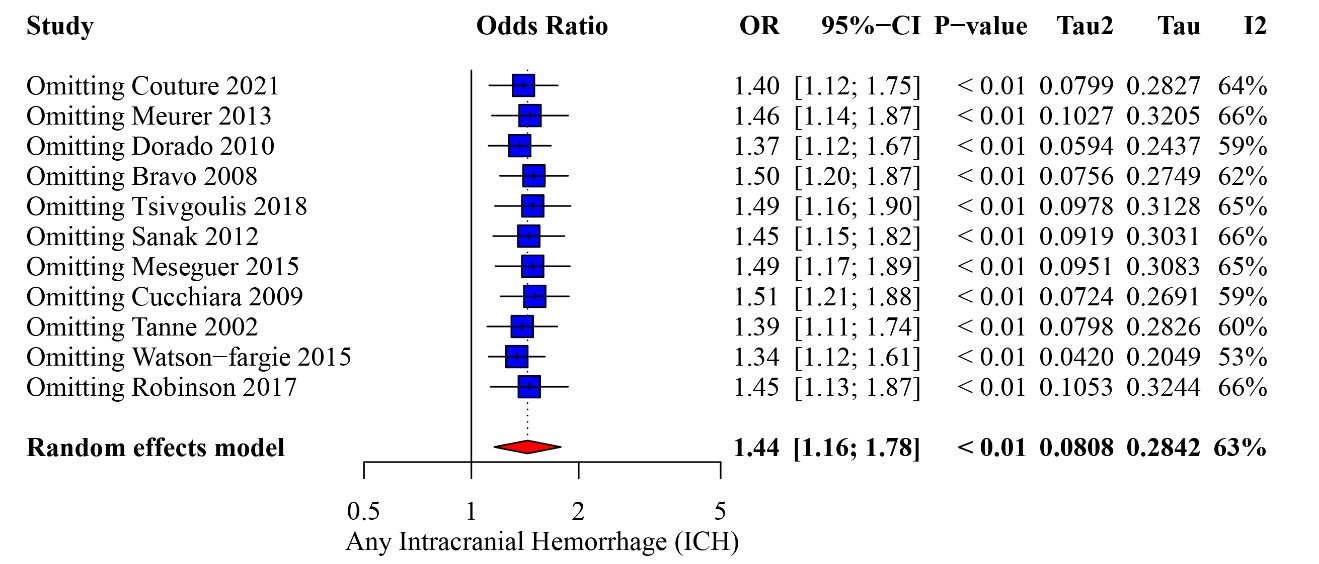


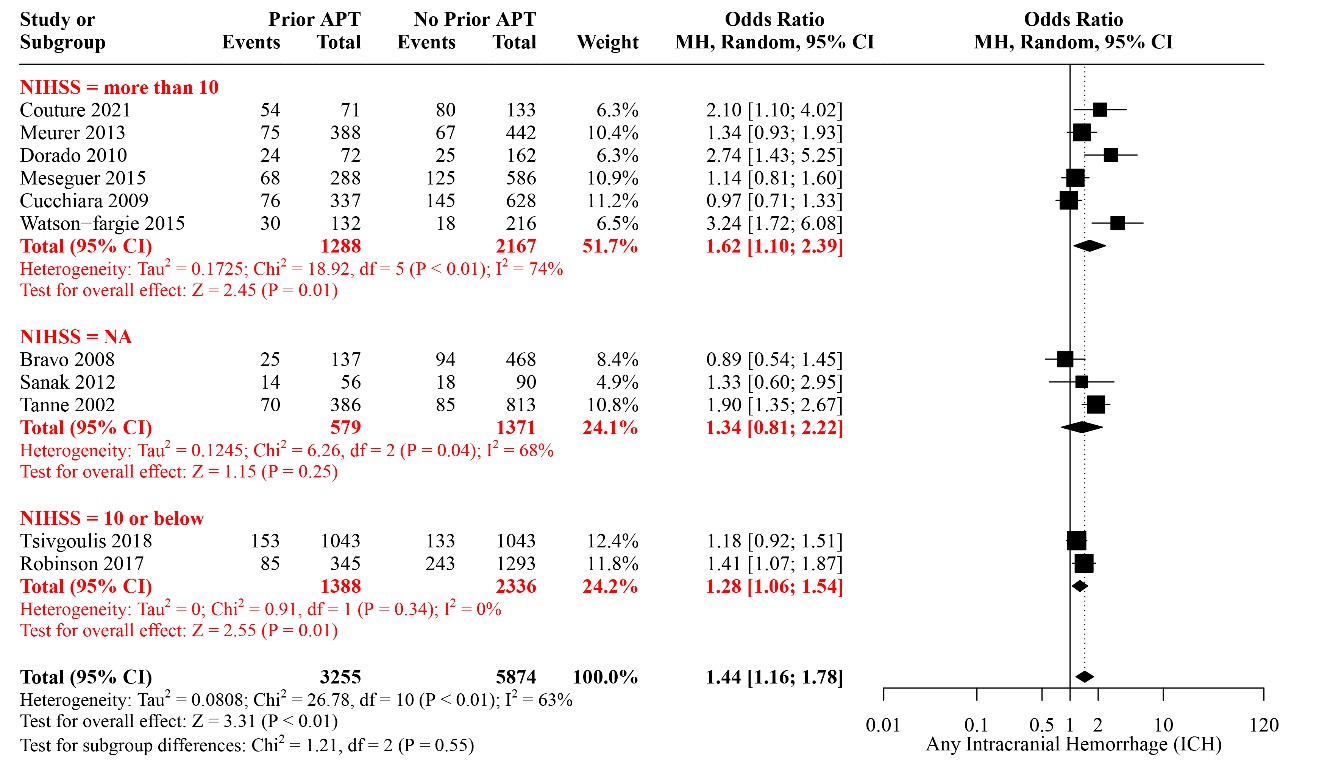
Figure S12: Forest plot of subgroup analysis for any ICH based on baseline NIHSS.

Figure S13: Forest plot of sensitivity analysis (Leave-One-Out test) for mortality outcome.


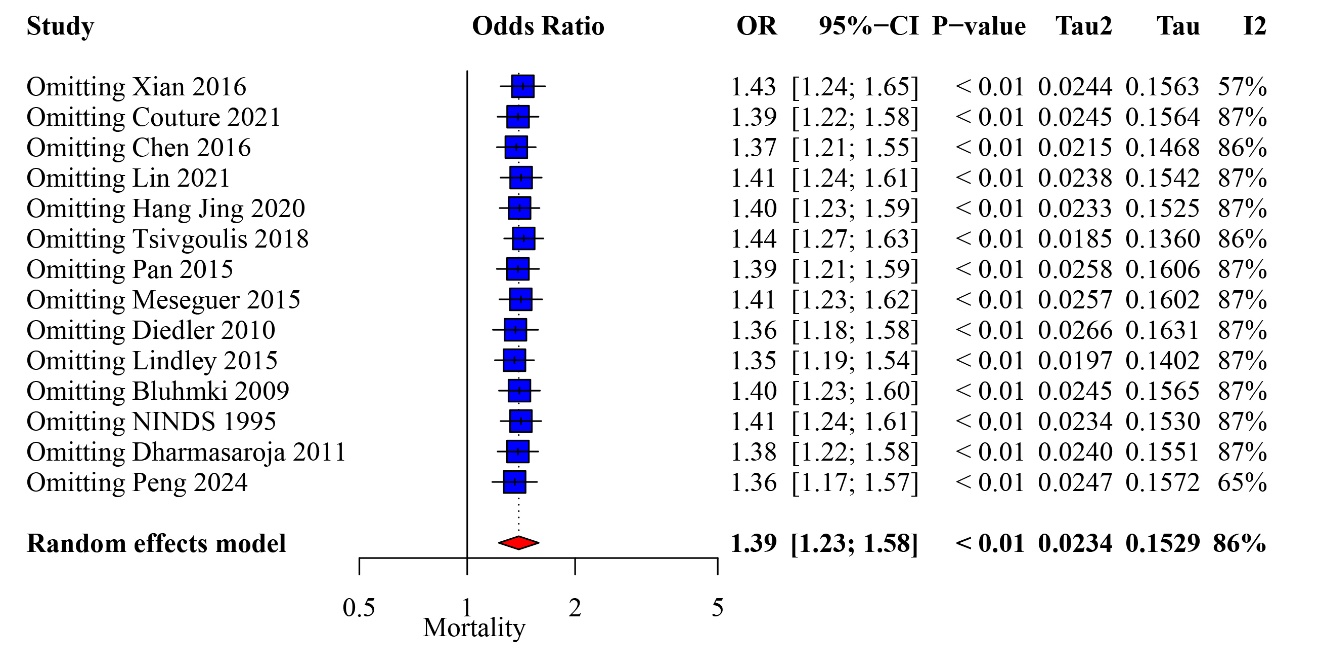


Figure S14: Forest plot of subgroup analysis for mortality outcome based on baseline NIHSS.


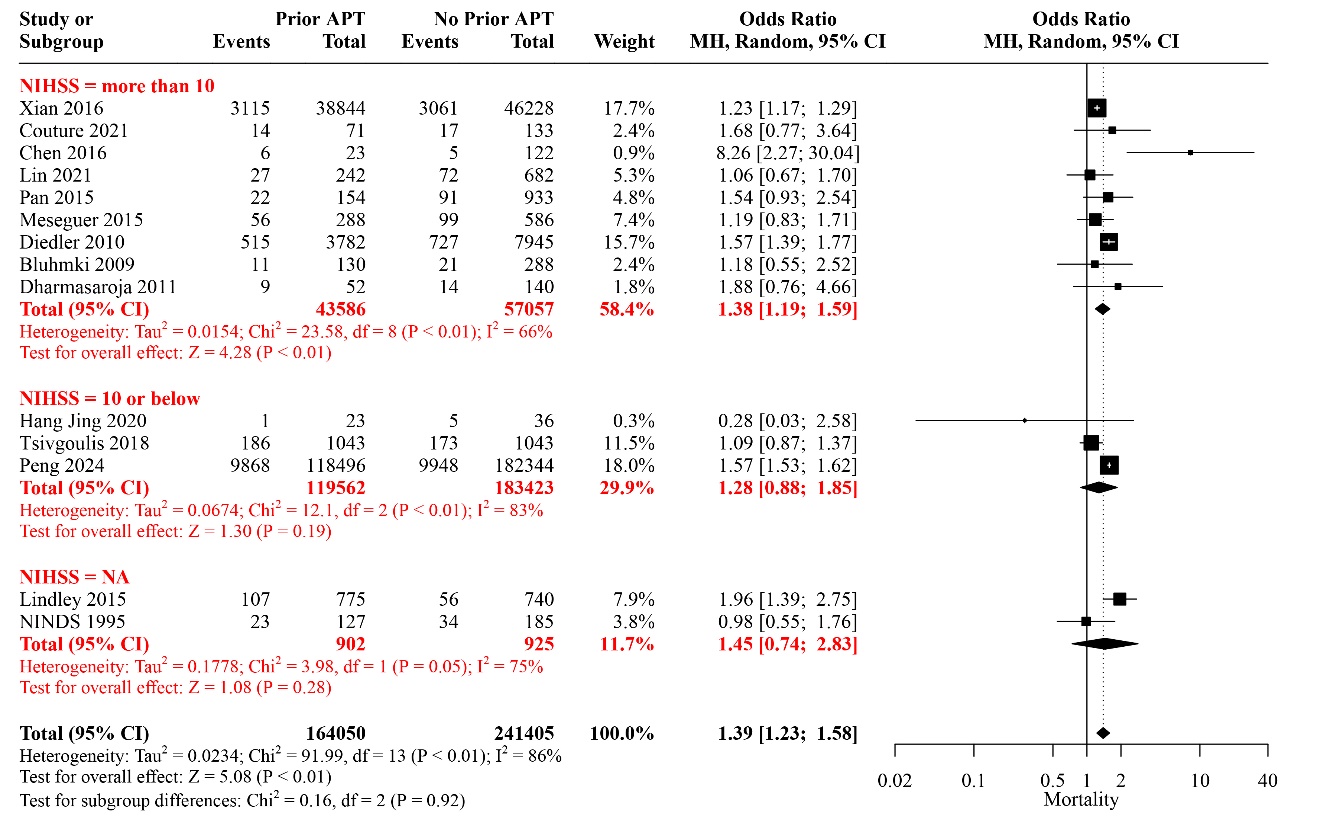


Figure S15: Forest plot of subgroup analysis for mortality outcome based on study design.


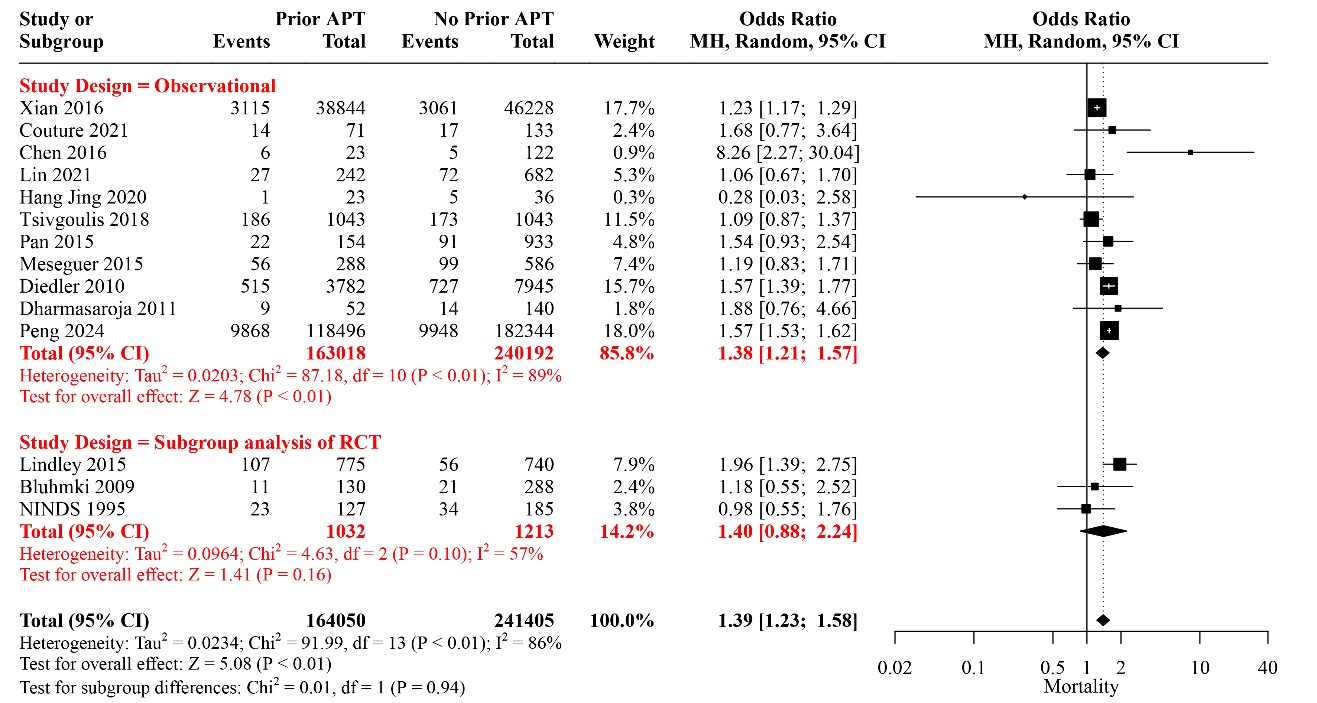


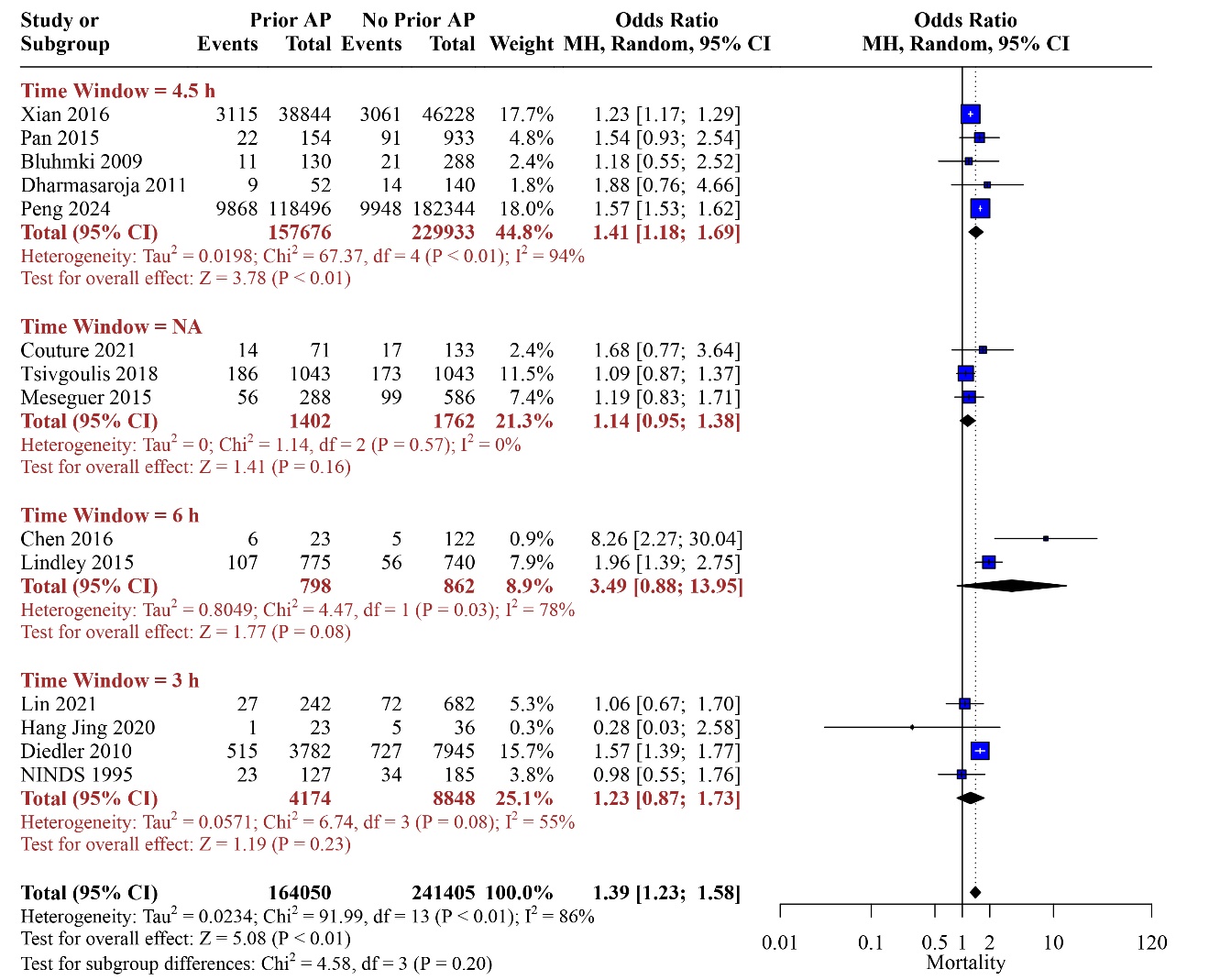
Figure S16: Forest plot of subgroup analysis for mortality outcome based on time window.

Figure S17: Forest plot of sensitivity analysis (Leave-One-Out test) for good functional outcome (mRS 0-2).


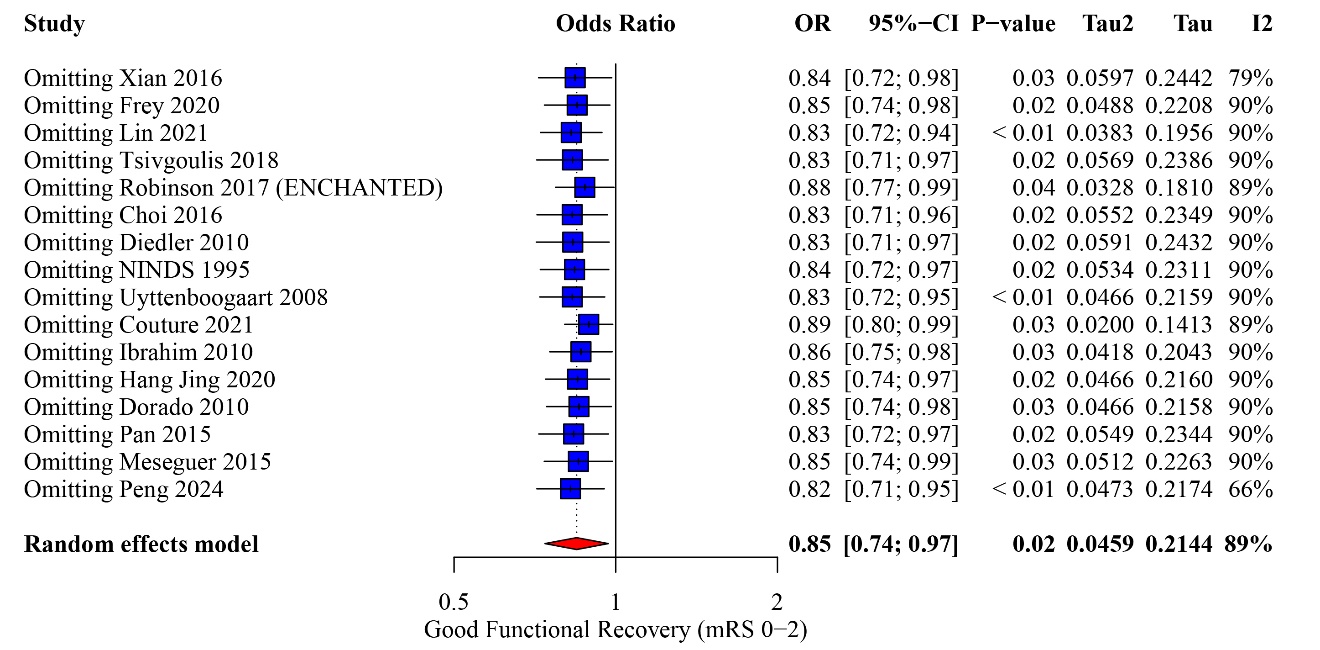


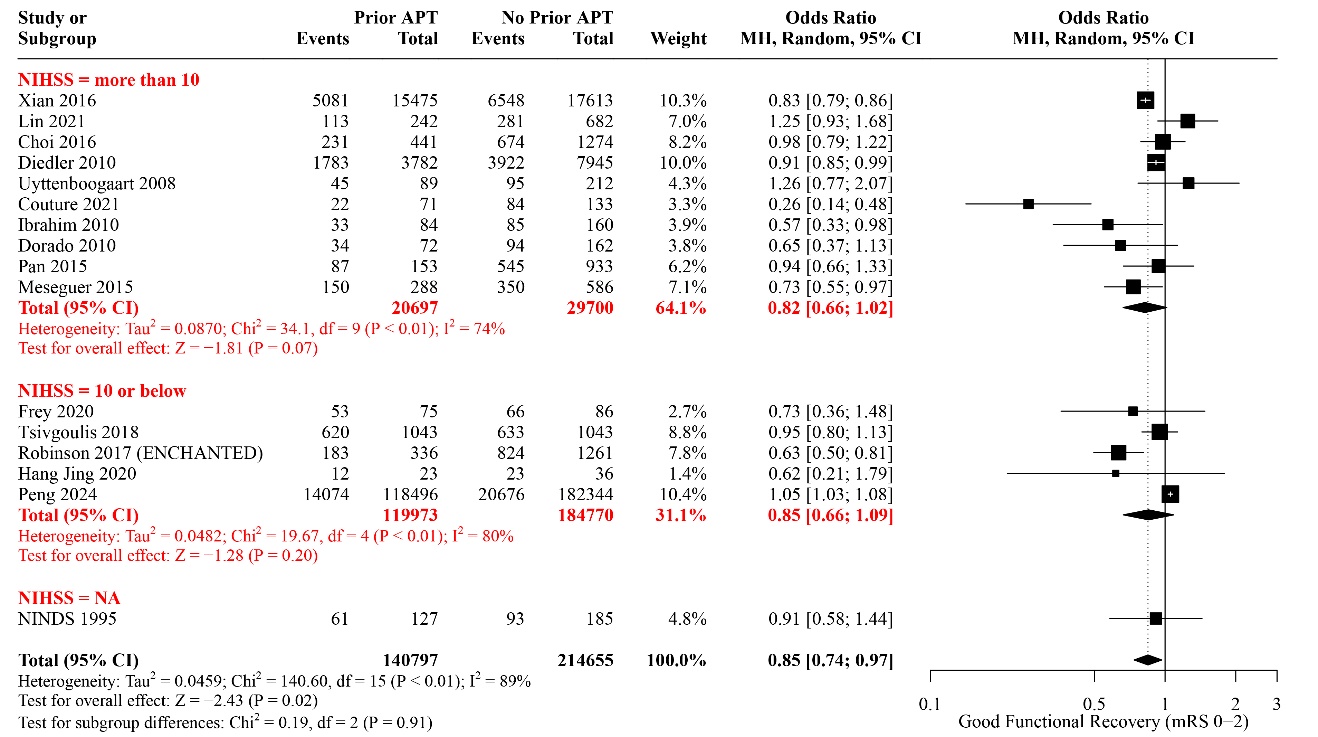
Figure S18: Forest plot of subgroup analysis for mRS 0-2 outcome based on baseline NIHSS.

Figure S19: Forest plot of subgroup analysis for mRS 0-2 outcome based on Study design.


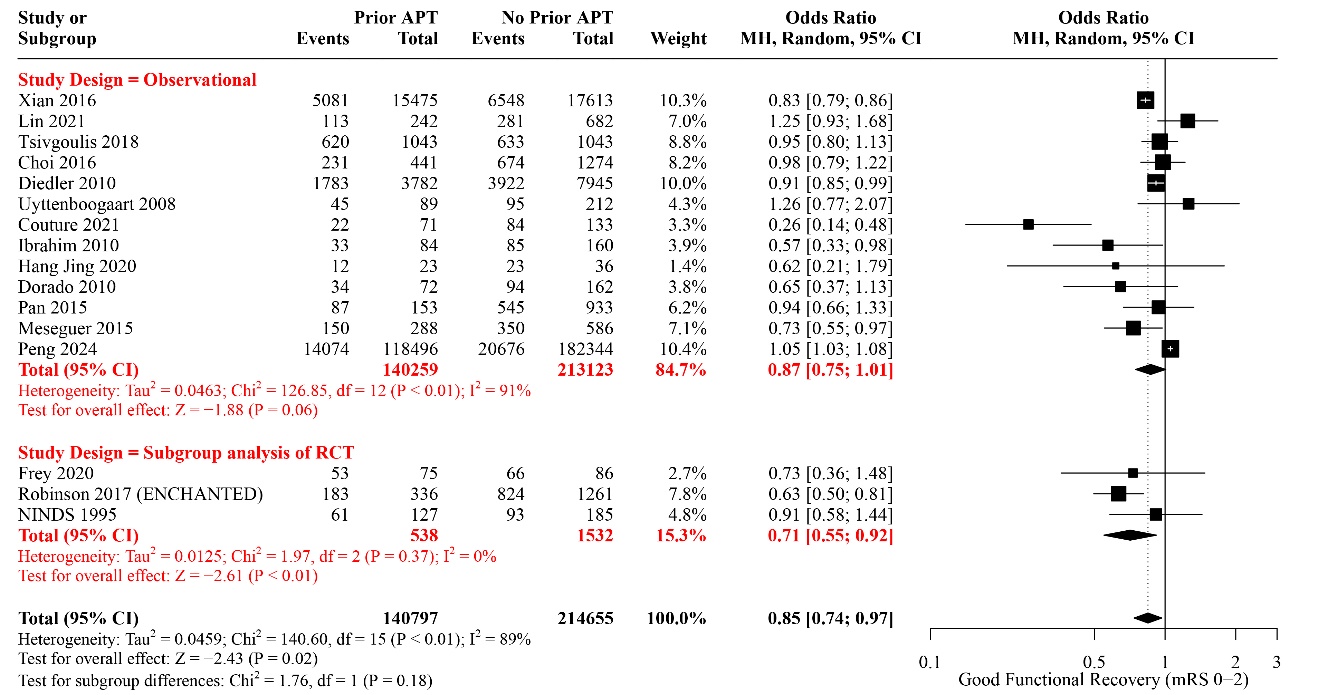


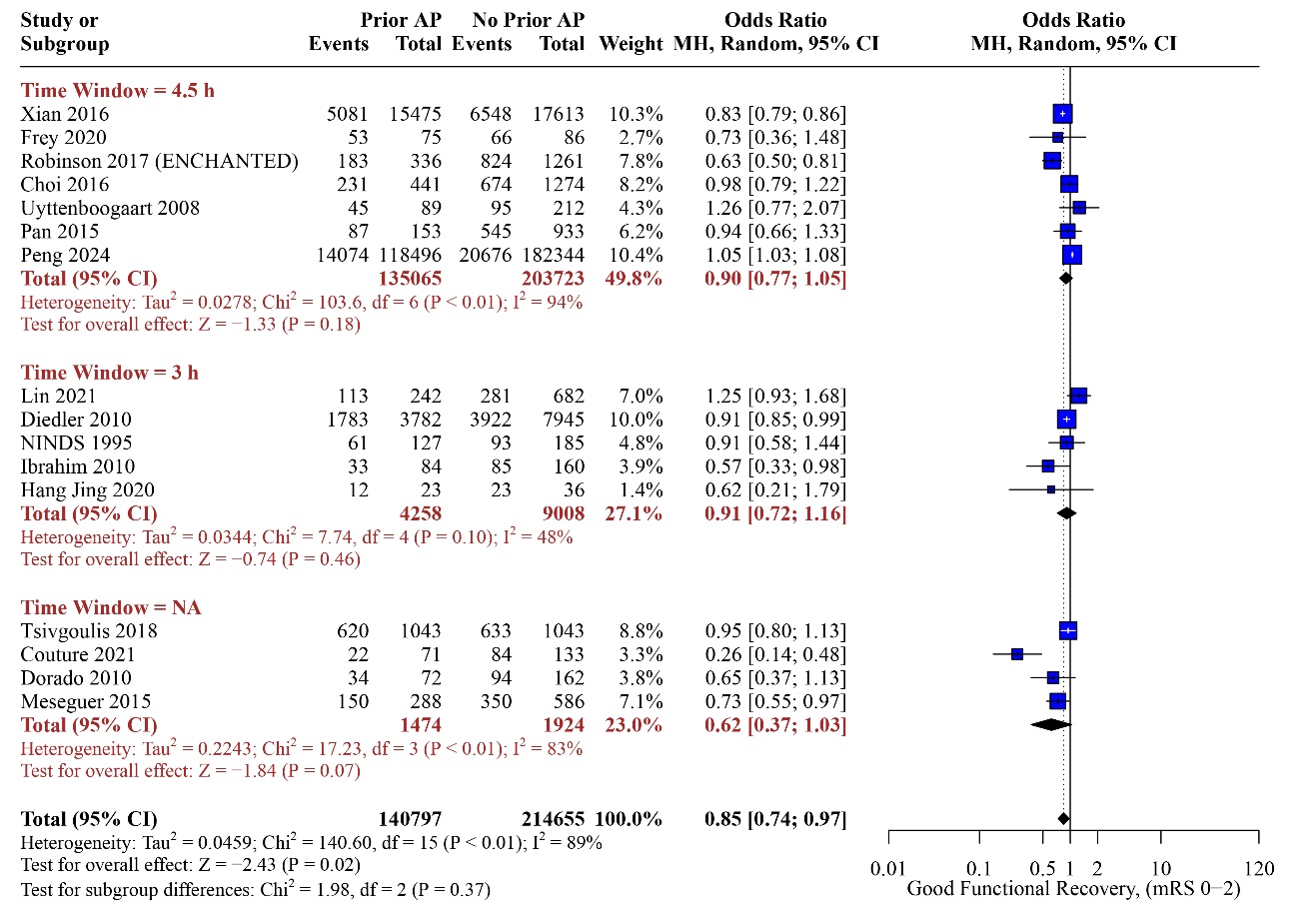
Figure S20: Forest plot of subgroup analysis for good functional recovery based on time window.

Figure S21: Forest plot of sensitivity analysis (Leave-One-Out test) for poor functional outcome (mRS 3-6).


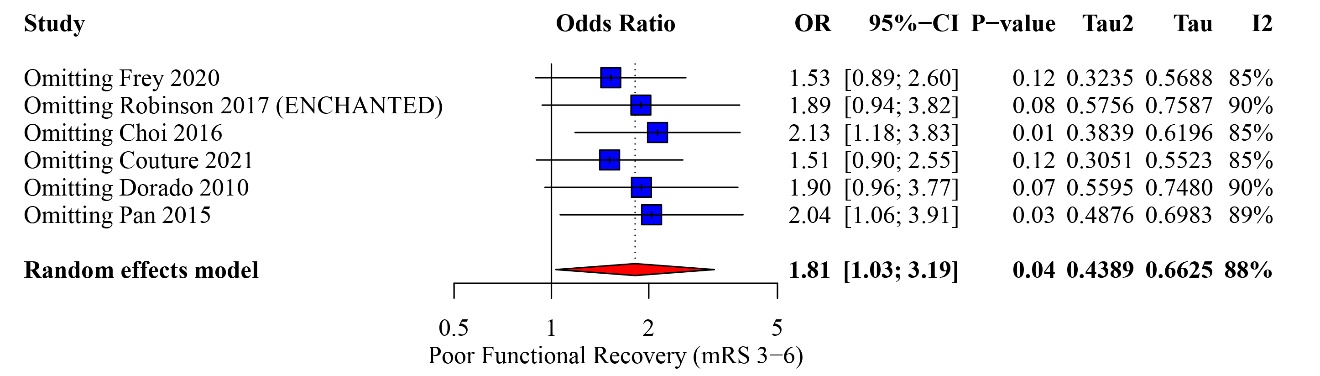


Figure S22: Forest plot of subgroup analysis for mRS 3-6 outcome based on baseline NIHSS.


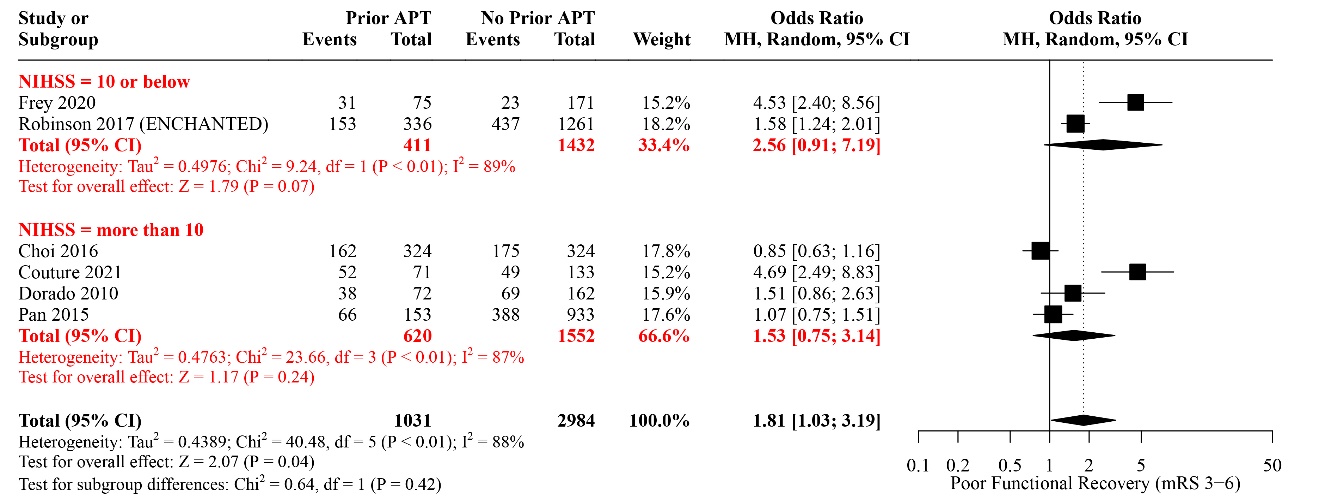


Figure S23: Forest plot of subgroup analysis for mRS 3-6 outcome based on study design.


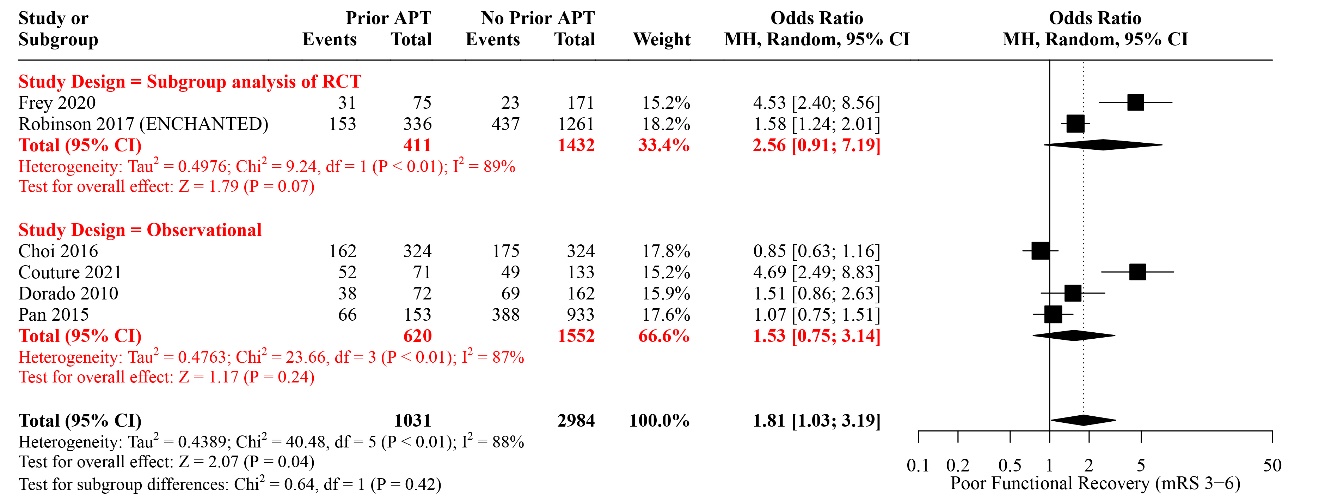

Supplement: Supplementary file 1 — Supplementary file1 (DOCX 6.14 MB) [file 10072_2025_8024_MOESM1_ESM.docx]
